# A meta-analysis of longitudinal studies on the interplay between sleep, mental health, and positive well-being in adolescents

**DOI:** 10.1016/j.ijchp.2023.100424

**Published:** 2023-12-02

**Authors:** Valeria Bacaro, Katarina Miletic, Elisabetta Crocetti

**Affiliations:** Department of Psychology “Renzo Canestrari”, University of Bologna, Italy

**Keywords:** Sleep, Adolescents, Mental health, Positive well-being, Meta-analysis

## Abstract

**Objective:**

This review aimed to summarize longitudinal research about the interplay between sleep, mental health, and positive well-being in adolescents.

**Method:**

Multiple search strategies were applied until 28^th^ January 2023 to identify relevant research published in peer-reviewed journal articles or available grey literature. A final set of 63 studies were included in the systematic review and 42 in the meta-analysis.

**Results:**

Results highlighted that long sleep duration, good sleep quality, and low insomnia symptoms were bidirectionally related to lower internalizing (Sleep T1 → Internalizing symptoms T2: *r* = -.20, *p* < .001; Internalizing symptoms T1 → Sleep T2: *r* = -.21, *p* < .001) and externalizing (Sleep T1 → Externalizing symptoms T2: *r* = -.15, *p* < .001; Externalizing symptoms T1→ Sleep T2: *r* = -.17, *p* < .001) symptoms, and to higher levels of psychological well-being (Sleep T1 → Psychological well-being T2: *r* = .15, *p* < .001; Psychological well-being T1 → Sleep T2: *r* = .15, *p* < .05). Moreover, good sleep was positively related to higher subjective well-being at a later time point (*r* = .18, *p* < .001).

**Conclusions:**

Overall, these findings suggest a bidirectional relation between different aspects of sleep, mental health, and positive well-being.

## Introduction

Adolescence is a period of substantial neurobiological, behavioral, and psychosocial changes (Meeus, 2016). The complex interplay between biological and brain maturation and environmental factors makes adolescence a moment of particular vulnerability to mental health issues and well-being (Blakemore, 2019). Sleep has a critical role in many psychological, cognitive, and behavioral processes that contribute to both mental health and positive well-being (Goldstein & Walker, 2014; Krause et al., 2017) and is also known to undergo an essential developmental change in adolescence (Carskadon, 2011). This study aims to systematically review the pathways connecting sleep, mental health, and positive well-being in adolescents.

### Mental health and well-being

Understanding how to promote mental health and well-being is both a research and a societal priority. While in the last century, the focus was mainly on its negative side (e.g., how to reduce anxiety and depressive symptomatology), in the last decades, a progressive shift toward the necessity to enhance its positive components has been undertaken (Keyes, 2002). This advancement was prompted by the World Health Organization (World Health Organization, 2020) and led to a multidisciplinary effort toward a positive definition of well-being.

Mental health in adolescents is typically assessed through *internalizing* (i.e., symptoms directed toward oneself, causing internal distress) and *externalizing symptomatology* (i.e., outer-directed problems that cause conflict between the individual and their environment), which has shown an increase in this population, with prevalence at a worldwide level estimated at around 13% (Achenbach et al., 2016, Polanczyk et al., 2015). Cumulative evidence has shown that these symptoms constitute a crucial risk factor for several adverse health consequences, such as suicide-related behaviors (Hartley et al., 2017), adulthood delinquency (Leschied et al., 2008), and lower academic achievement (Okano et al., 2019).

Regarding positive well-being, two main theoretical frameworks have been proposed for investigating this phenomenon. The first is focused on the concept of hedonic well-being (i.e., positive emotional states derived from satisfying an individual's physical, intellectual, or social needs), referred to as *subjective well-being* (Diener, 1984). Subjective well-being includes an affective component (i.e., a balance between positive and negative affect) and a cognitive component (i.e., judgments concerning one's life satisfaction) (Ryan & Deci, 2001). The second has at its core the concept of eudaimonic well-being (i.e., the process of manifesting one's potential, particularly as it relates to one's goals, personal growth, and purpose in life), defined as a state of psychological well-being explicitly connected with the individual's self-realization (Ryff and Singer, 2006). In adolescents, on the one hand, young people experience typical emotional instability, and their psychological well-being can be hampered by the necessity to cope with multiple developmental tasks; on the other hand, optimal levels of subjective and psychological well-being can help prevent and mitigate the adverse effects of stressful life events against developing mental health issues (Park et al., 2004). In conclusion, it is essential to adopt a dual perspective, considering both sides of adolescents' positive development, focusing on which factors can prevent mental health issues and promote positive well-being.

### Sleep, mental health, and positive well-being in adolescence

Sleep is a fundamental human psychophysiological function, and good sleep quality is essential in adolescents' healthy development. Nevertheless, a trend of sleep disruption and irregular healthy habits in adolescents was highlighted (Jakobsson et al., 2020). Specifically, according to the "Perfect Puberty Storm" model (Carskadon, 2011), adolescence is a period in which quantitative (i.e., the number of hours spent sleeping per night) and qualitative (i.e., the subjective satisfaction with sleep, regular sleep schedule, a short length of time necessary to fall asleep, and adequate sleep maintenance) aspects of sleep are influenced by the simultaneous occurrence of numerous biological, psychosocial, and environmental changes that often come in conflict with each other. Developmental changes in the circadian rhythm system (i.e., organism's physiological and biological processes every 24 hours), together with the adolescents' increasing needs for autonomy and social connection, cause a natural delay in bedtime, which contrasts with societal demands for students to wake up earlier than before (Carskadon, 2001, Carskadon, 2011). This confluence of factors can result in a trend of a shift from morningness (i.e., a preference to get up and go to bed early) towards eveningness (i.e., a preference to get up and go to bed lately) during adolescence, which can, in turn, lead to sleep deprivation and sleep difficulties since its discordance with their school and social schedules (Fischer et al., 2017; Bacaro et al., 2023). This is particularly concerning considering the well-established connections between sleep difficulties and increased vulnerability to mental health problems, often emerging in adolescence (Harvey et al., 2011).

Sleep difficulties and insomnia symptoms (i.e., repeated difficulties with sleep initiation and maintenance despite having an adequate opportunity to sleep, resulting in significant distress and daytime consequences) were traditionally considered secondary psychopathology symptoms of major disorders (e.g., depressive and anxiety disorders). Nowadays, these difficulties are considered a transdiagnostic process that contributes to a plethora of mental health issues (Harvey et al., 2011). Interactions between such processes could explain the multifaceted and often bidirectional connections between sleep and both mental health and positive well-being.

#### Sleep and internalizing symptomatology

In terms of quantity or quality, poor sleep is linked to emotional difficulties, depression, and anxiety symptomatology (Becker et al., 2015; Lovato & Gradisar, 2014; Marino et al., 2021). Depressive rumination and the excessive vigilance found in anxiety sufferers are commonly proposed as mechanisms contributing to sleep difficulties, exacerbating internalizing symptomatology through poorer emotional processing (e.g., Blake et al., 2018). While evidence points towards a bidirectional influence between sleep and internalizing psychopathology, different studies show contradictory results, and the heterogeneity of the currently available data does not allow for definite conclusions (Becker et al., 2015; Baglioni et al., 2011).

#### Sleep and externalizing symptomatology

Insufficient or inadequate sleep has been connected to adolescents' externalizing symptoms such as addictions, impulsivity, and risky and antisocial behaviors (Becker et al., 2015; Tarokh et al., 2016; Womack et al., 2013). Moreover, impulsive behaviors (e.g., substances used as sleeping aids) can contribute to the development of addictions (e.g., Brower et al., 2001). Though a large part of these results has been attributed to difficulties in cognitive-emotion processing caused by insufficient or poor quality sleep (Krizan & Herlache, 2016), such as amplified reward sensitivity (Goldstein & Walker, 2014), some longitudinal studies (Kortesoja et al., 2020) have found connections in directions opposite from those anticipated. Thus, it is of utmost importance to understand the bidirectionality of this relation to clarify the nature of this potential vicious circle.

#### Sleep and subjective well-being

Sleep is hypothesized to be bidirectionally connected to both the affective and cognitive facets of subjective well-being. For what concern the affective aspect, on the one hand, poor sleep can lead to less efficient affect regulation, and on the other hand, heightened emotional reactivity can be associated with poor sleep (Reddy et al., 2016). Furthermore, regarding the cognitive aspect, sleep disruption can be associated with a negative perception bias that may also explain the connection between poor sleep and adolescents' more negative perceptions of quality of life (Williamson et al., 2021). However, it is equally possible that objectively worse living conditions lead to both lower life satisfaction and poorer sleep. Understanding the role of affective and cognitive aspects in the relation between sleep and subjective well-being can be relevant to studying positive well-being and psychopathology.

#### Sleep and psychological well-being

There is a particular dearth of longitudinal studies attempting to establish temporal precedence between sleep and psychological well-being. Psychological well-being includes several aspects related to adolescents’ sleep (e.g., autonomy, resilience, self-esteem, and self-regulation) (Ryff, 2014)*.* For example, sleep patterns and disturbances can be related to an impairment in the functioning of the prefrontal cortex, which can interfere with the ability to regulate behaviors and feelings (Dahl, 1996). These two phenomena seem to share some crucial processes for adolescent health. Nevertheless, most studies focused on the relation between sleep and mental health. Still, a comprehensive understanding of the longitudinal interplay between sleep and positive aspects of functioning, such as psychological well-being, is needed.

### The present study

**Fig. 1:**
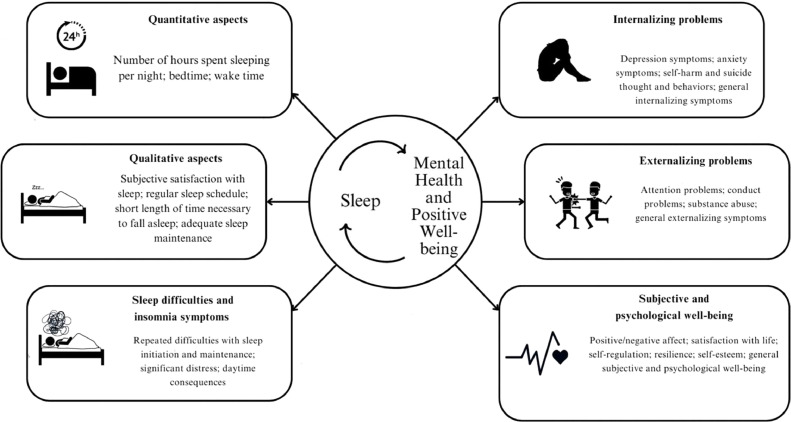
Overview of the main dimensions

The simultaneous abundance and lack of conclusiveness of the data seeking to explain the connections between sleep, mental health, and positive well-being variables point to a need for a systematic synthesis of the available information. While some meta-analyses have already been conducted (e.g., Alvaro et al., 2013; Short et al., 2020), few so far have focused exclusively on longitudinal data in the adolescent period, and all have focused on only a few mental health and positive well-being variables at a time. Because adolescence is a time of significant changes in both sleep and psychological characteristics, and because of sleep's transdiagnostic influence on many mutually interacting mental health and positive well-being phenomena, it is essential to provide a systemic overview of longitudinal data about the reciprocal connections between sleep and a wide range of mental health and positive well-being phenomena in adolescents (for an overview of the main dimensions considered see Fig. 1).

## Method

The study is part of a larger project examining the bidirectional link between sleep and adolescent psychosocial development (PROSPERO preregistration ID: CRD42021281002). It was performed according to the PRISMA (Preferred Reporting Items for Systematic Reviews and Meta-Analyses) guidelines (Page et al., 2021) (See Document S1 in the supplemental material for the PRISMA checklist). Two independent raters independently screened the titles and abstracts, examined the full texts, and extracted data for analysis from the selected studies (Crocetti, 2016). Disagreements in the evaluations by the two judges were resolved through discussion with a third rater. The searches and the screening process were managed on Citavi 6 software.

### Literature search and eligibility criteria

A literature search strategy was implemented in order to identify pertinent research published in peer-reviewed journal articles or accessible as grey literature until January 28, 2023. The full search strategy is reported in Document S2 of the supplemental material. Studies were considered eligible if they: (a) evaluated adolescents from the general population aged 10-19 years; (b) involved a longitudinal study design; (c) assessed at least one sleep indicator through objective (e.g., actigraphy) or subjective (e.g., sleep diaries, questionnaires) instruments; (d) assessed at least one aspect of mental health and positive well-being (for the categorization of the variables see Fig. 1). Both peer-reviewed journal articles and grey literature that can be retrieved through database searches (e.g., doctoral dissertations) were included, and no restrictions were applied based on the year and the language of publication (when references were published in a language other than English, professional translators were contacted).

### Selection and coding of primary studies

**Fig. 2:**
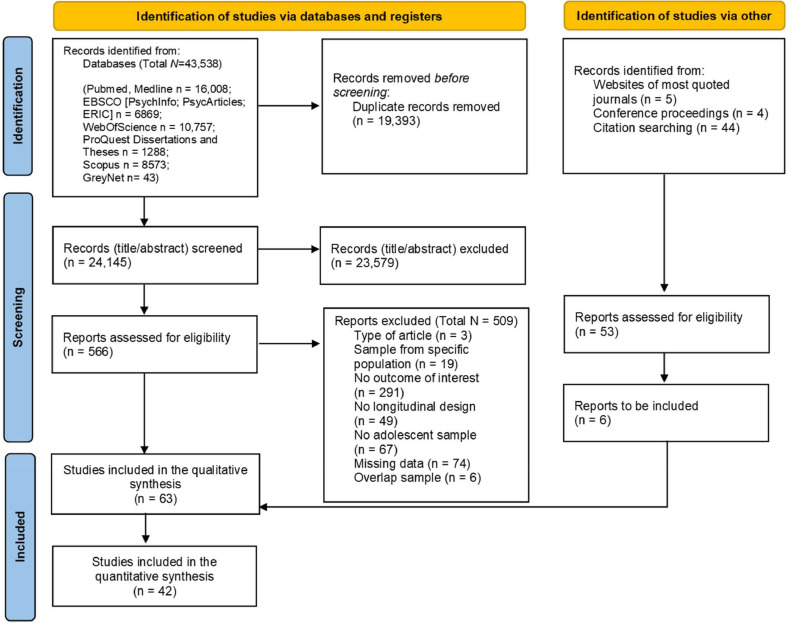
PRISMA diagram search flow

Fig. 2 reports the detailed flow chart of the selection process. Titles and abstracts were screened (substantial percentage of agreement, Cohen's Kappa = 0.77). Next, 566 full-texts were examined (high percentage of agreement; Cohen's Kappa = 0.71). In total, 63 studies were included in the systematic review, with 42 also included in the meta-analytic calculations. The data extraction of the included studies was performed following a coding protocol (the complete list of variables extracted and the procedure observed are reported in the supplemental materials, Document S3). The percentage of agreement was high (i.e., 91.8%).

### Strategy of analysis

All the statistical analyses were conducted using the software Prometa 3 to estimate how sleep variables measured at one time point (e.g., sleep duration at T1) were related to mental health and positive well-being variables at the last time point considered in the primary studies (T2) and mental health and positive well-being variables at one time point (e.g., internalizing problems at T1) were linked to sleep at the last available time point (T2). For the full analysis strategy, see Document S4 of the Supplemental materials.

## Results

### Meta-analyses of the longitudinal interplay between sleep, mental health and positive well-being

Sixty-three studies were included in the systematic review; the main characteristics are reported and discussed in Document S5 of the Supplemental materials. All the effect sizes of the included studies are reported in the supplemental materials (Document S6). Results of all the meta-analyses for the longitudinal interplay between sleep, mental health and positive well-being are reported below. All forest plots are reported in the supplemental materials.

#### The interplay between sleep and internalizing symptoms

Regarding the interplay between sleep and internalizing symptoms (i.e., depression and anxiety symptoms, self-harm and suicide intention, and general internalizing symptoms), 46 studies examined this link either unidirectionally or bidirectionally (*n*=21). Regarding the longitudinal association between sleep at one time point (T1) and internalizing symptoms at a later time (T2), 26 studies were included in the meta-analytic results. Results of the meta-analysis (Table 1) showed a significant moderate association between longer sleep duration, higher sleep quality, and lower presence of insomnia symptoms with lower internalizing symptoms over time (*r* = -.20, *p* < .001, forest plot Document S7). Heterogeneity statistics were high and significant. Moderator analyses highlighted no moderation by the mean age (*B* = -.30, *p* = .67) or by the time lag between waves (*B* = -.23, *p* = .54). Results were moderated by the method of sleep assessment (*Q* = 18.5, *p* < .001). Specifically, the association was stronger in studies that used subjective measures for assessing sleep. Furthermore, no publication bias was detected from the visual investigation of the funnel plot and confirmed by non-significant Egger's test (*p* = .47). Results of the studies not included in the meta-analysis confirmed this association, except for one study (Liu et al., 2021a) which found no association between daytime sleepiness and internalizing symptoms over time.

**Table 1:** Overall Meta-Analytic Calculations of the Bidirectional Relations Between Sleep, Mental Health, and Positive Well-being

Overall effect Between Sleep T1 → Mental Health and Positive Well-being T2^1^	*k*	ES [95% CI]	*Q*	I^2^	Egger's test	Overall effect Between Mental Health and Positive Well-being Variables T1 → Sleep T2^2^	*k*	ES [95% CI]	*Q*	I^2^	Egger's test
**Sleep T1 → Internalizing Symptoms T2**	26	*r* = -.20⁎⁎ [-.24, -.16]	275.54⁎⁎⁎	90.93	0.99	**Internalizing Symptoms T1 → Sleep T2**	24	*r* = -.21⁎⁎⁎ [-.29, -.14]	2005.73	98.	-5.00
**Sleep T1 → Externalizing Symptoms T2**	12	*r* = -.15⁎⁎⁎ [-.19, -.11]	48.49⁎⁎⁎	77.32	-0.65	**Externalizing Symptoms T1 → Sleep T2**	6	*r* = -.17⁎⁎⁎ [-.26, -.09]	62.28*⁎⁎	91.97	-2.62
**Sleep T1 → Subjective Well-being T2**	6	*r* = .18⁎⁎⁎ [.09, .28]	36.59⁎⁎⁎	86.33	-2.66						
**Sleep T1 → Psychological Well-being T2**	6	*r* = .15⁎⁎⁎ [.11, .19]	16.60⁎⁎	69.89	1.58	**Psychological Well-being T1 → Sleep T2**	7	*r* = .15* [.03, .28]	344.42⁎⁎⁎	98.26	10.46

*Notes*. ^1^Cross-lagged effects between sleep measured at one time point (T1) and mental health and positive well-being variables measured at the last time point (T2) considered in the study. ^2^Cross-lagged effects between mental health and positive well-being variables measured at one time point (T1) and sleep measured at the last time point (T2) considered in the study. *k* = number of studies; ES = Effect Size; *Q* = heterogeneity test; I^2^ = heterogeneity estimate. ^⁎⁎⁎^*p* <.001

⁎⁎ *p* < .01

⁎ *p* < .05.

Examining the opposite direction, 27 studies focused on the association between internalizing symptoms at one point (T1) and sleep variables at a later time (T2); of these, three studies did not report the needed effect size. Meta-analytic results (Table 1, forest plot Document S8) showed a significant effect (*r* = -.21, *p* < .001), highlighting that higher internalizing symptoms were associated with longer sleep duration, lower sleep quality, and higher presence of insomnia symptoms over time. Heterogeneity statistics were high and significant. This result was not moderated by participants' age at T1 (*B* = -.11, *p* = .66) or by the time lag between waves (*B* = -.26, *p* = .28) and was not affected by publication bias, as evident from the visual investigation of the funnel plot and the non-significant Egger's test (*p* = .11). Results of the studies not included in the meta-analysis confirmed this association.

#### The interplay between sleep and externalizing symptoms

Regarding the interplay between sleep and externalizing symptoms (i.e., attention problems, conduct problems, substance abuse, general externalizing symptoms), 14 studies examined this link, of which six of them bidirectionally. Regarding the longitudinal association between sleep at one time point (T1) and externalizing symptoms at a later time (T2), 12 studies were included in the meta-analytic results, while two did not report the needed effect size. Results (See Table 1, Document S9 for the forest plot) showed a significant effect (*r* = -.15, *p* < .001). Specifically, longer sleep duration, higher sleep quality, and lower presence of insomnia symptoms were associated with lower externalizing symptoms over time. Heterogeneity statistics were high and significant. The results were not moderated by the mean age of participants (*B* = -.31, *p* = .27) but were moderated by the amount of time lag between waves (*B* = -.25, *p* = .01, the effect decreases with the increase of time lag between waves). Furthermore, the visual investigation of the funnel plot showed that this result was not affected by publication bias (Egger's test, *p* = .89). Regarding the studies not included in the meta-analysis, one confirmed these results (Liu et al., 2023), while the other (Wong et al., 2015) showed no association between insomnia symptoms and externalizing symptoms over time.

Examining the opposite direction, six studies focused on the association between externalizing symptoms at one point (T1) and sleep variables at a later time (T2). Therefore, a meta-analysis was conducted (Table 1, Document S10 for the forest plot), and results showed a significant small-to-moderate association (*r* = -.17, *p* < .001) between higher externalizing symptoms and shorter sleep duration, poorer sleep quality, and higher insomnia symptoms over time. Heterogeneity statistics were high and significant. This result was not moderated by participants' age at T1 (*B* = .28, *p* = .28) or by the time lag between waves (*B* = -.40, *p* = .26) and was not affected by publication bias (Egger's test, *p* = .35).

#### The interplay between sleep and subjective well-being

Regarding the interplay between sleep and subjective well-being (i.e., positive and negative affects; life satisfaction, and general subjective well-being), seven studies examined this link unidirectionally or bidirectionally (*n*=2). Regarding the longitudinal association between sleep at one time point (T1) and subjective well-being at a later time (T2), six studies were included in the meta-analytic results, while one reported only qualitative information. Results (Table 1, Document S11 for the forest plot) showed a significant association (*r* = .18, *p* < .001) between longer sleep duration, higher sleep quality, and lower insomnia symptoms with higher subjective well-being over time. Heterogeneity statistics were high and significant. Results were not moderated by the mean age of participants (*B* = -.12, *p* = .78) or by the time lag between waves (*B* = .29, *p* = .70). Furthermore, the visual investigation of the funnel plot showed that this result was not affected by publication bias, as evident from the non-significant Egger's test (*p* = .33). The result of studies not included in the meta-analysis confirmed this association (Kenny et al., 2016) also suggesting potential bidirectionality of these results (Genta et al., 2021, Yip et al., 2022).

#### The interplay between sleep and psychological well-being

Regarding the interplay between sleep and psychological well-being (i.e., self-regulation, self-esteem, resilience), nine studies examined this association unidirectionally or bidirectionally (*n*=5). Regarding the longitudinal link between sleep at one time point (T1) and psychological well-being at a later time (T2), seven studies were included in the meta-analytic results, while one did not report the needed effect size. Results (Table 1, Document S12 for the forest plot) showed a significant association between longer sleep duration, higher sleep quality, and lower insomnia symptoms with higher psychological well-being over time (*r* = .15, *p* < .001). Heterogeneity statistics were moderate and significant. Despite that, moderator analyses highlighted that results were not moderated by the mean age of participants (*B* = -.25, *p* = .22) or by the time lag between waves (*B* = .14, *p* = .78). Furthermore, the visual investigation of the funnel plot showed that this result was not affected by publication bias, (Egger's test, *p* = .57). The result of the study not included in the meta-analysis confirmed this association (Roberts et al., 2002).

Examining the opposite direction, seven studies focused on the association between psychological well-being at one point (T1) and sleep variables at a later time (T2). Results (Table 1, see Document S13 for the forest plot) showed a significant effect (*r* = .15, *p* < .05) for which higher level of psychological well-being were associated with longer sleep duration, higher sleep quality, and lower presence of insomnia symptoms. Heterogeneity was high and significant. The results were not moderated by participants' age at T1 (*B* = -.36, *p* = .49) or by the time lag between waves (*B* = .13, *p* = 80), and were not affected by publication bias, as evident from the non-significant Egger's test (*p* = .88).

#### The role of circadian preference

Four studies evaluated the link between the circadian preference of adolescents and different mental health outcomes. Given their heterogeneity in considering different mental health indicators and the missing needed effect size for meta-analytic calculations, only a qualitative review of the findings was conducted (see Document S6 in the supplemental materials). Results showed that the morning circadian preference of adolescents at one time was associated with lower depression and anxiety symptoms at a later time (Alvaro et al., 2017; Haraden et al., 2017). This result was not confirmed by the other study that evaluated this link (Li et al., 2021), which highlighted no association between morning type circadian preference and internalizing symptoms over time. Moreover, one study found an association between morning type circadian preference and self-efficacy over time (Scherrer & Preckel, 2021).

## Discussion

During adolescence, a chronic reduction in sleep duration, a disruption in sleep quality, and a delay of circadian preference are observed and attributed to a combination of physiological and psychosocial phenomena characteristics of this developmental period. These changes place them at a greater risk of experiencing higher mental health problems and lower well-being, which, in turn, can lead to a vicious circle of impairment of different aspects of adolescents' health. For these reasons, the present systematic review with meta-analysis aimed to extend prior knowledge, focusing on longitudinal studies examining the interplay between adolescents' sleep, mental health, and positive well-being. Results highlighted a bidirectional association between these two variables over time. The main findings in both directions are discussed below, suggesting future lines of research emphasizing the importance of taking a multifaceted approach to gain a comprehensive understanding of the phenomenon.

### Unveiling the dynamic interplay between sleep and internalizing symptoms

This systematic review with meta-analysis examined the longitudinal bidirectional relation between sleep and internalizing symptoms considering depression and anxiety symptoms, self-harm and suicidal intention, and general internalizing symptoms. Results showed a moderate bidirectional association between good sleep parameters (i.e., longer sleep duration, adequate sleep quality, and lower insomnia symptoms) and lower internalizing symptoms over time. These results are in line with previous literature (e.g., O'Callaghan et al., 2021, McMakin and Alfano, 2015).

Different biological, cognitive, behavioral, and psychosocial mechanisms were identified to explain this association (for a review, see Blake et al., 2018). Regarding biological processes, the responses to sleep difficulties increase cortisol and hypothalamic–pituitary–adrenal (HPA) axis dysregulation (van Dalfsen & Markus, 2018), which was highlighted to potentially contribute to developing anxiety and depression in adolescence. Similarly, increased cortisol reactivity in stressful situations was identified as a biological substrate of depression, which can lead to heightened emotional reactivity and difficulties in stressful situations that can, in turn, be associated with worsening sleep and insomnia symptoms (Asarnow, 2020). Moreover, cognitive and behavioral processes can play a role in this interplay. Regarding the cognitive ones, worry and rumination processes (i.e., thinking about personal concerns in an unproductive and repetitive way; Verstraeten et al., 2011) were found to be related to depression and anxiety symptoms in adolescents. For what concerns behaviors, a sedentary lifestyle or less physical activity affects sleep hygiene (Wang & Boros, 2019), and a lack of exposure to daylight may also impact sleep duration and quality (Keller et al., 2017). Moreover, some internalizing disorders may lead to less accurate self-reporting on subjective sleep measures (Bertocci et al., 2005). Finally, adolescent insomnia symptoms can be associated with impaired social interactions, exacerbating the emotional consequences of interpersonal rejection and impairing the ability to cope with daily interpersonal events. Concluding, sleep, mood, and arousal show overlapping regulatory systems, leading to the cycle for which the dysregulation in one system can negatively impact the others, so sleep disruption during adolescents may provide a pathway toward later affective dysregulation and vice versa (e.g., Dahl, 1996). For these reasons, adolescence represents a critical developmental window for understanding the mechanisms underlying the association between insomnia and internalizing symptoms to inform early intervention and prevention.

### Shedding light on the dynamic interplay between sleep and externalizing symptoms

This comprehensive review and meta-analysis explored the reciprocal longitudinal relation between sleep and externalizing symptoms, including attention and conduct behavior problems, substance abuse, and general externalizing symptoms. The findings revealed a moderate bidirectional association between good sleep and lower externalizing symptoms over time. These findings are consistent with prior research and meta-analyses (e.g., Becker et al., 2015; Pieters et al., 2014) and can be explained by the fact that externalizing symptoms and sleep share common neurobiological and neurocognitive characteristics (Krause et al., 2017; Lunsford-Avery et al., 2020). On the one hand, the ability to regulate emotions and control impulsive behaviors, which is significantly associated with externalizing symptomatology, was found to be impaired by poor sleep (Telzer et al., 2015). On the other hand, it was suggested that poor impulse control is related to irregular sleep routines and heightened arousal, creating a vicious circle. This systematic review highlighted the potential existence of a two-way relationship between sleep and externalizing behavior. These results can enhance the understanding of this reciprocal relation in adolescence, suggesting that addressing both sleep-related difficulties and externalizing behavioral symptoms, rather than focusing on either alone, could be crucial in the clinical practice to effectively manage the harmful cycle of sleep-mental health problems.

### Exploring the reciprocal association between sleep and positive well-being

This review also focused on the reciprocal association between sleep and positive well-being (i.e., subjective and psychological well-being). Few included studies investigated this link compared to the studies that evaluated the association between sleep and mental health outcomes. Results showed a small-to-moderate association between good sleep and higher subjective well-being over time. This aligns with the mechanism for which sleep disruption, in terms of quantitative and qualitative aspects, leads to emotional, behavioral, and attentional dysregulation, negatively affecting life satisfaction and mood, and this dysregulation, in turn, is likely to adolescents' sleep over time (Brown et al., 2017). Furthermore, results showed a bidirectional and small-to-moderate association between good sleep and higher levels of psychological well-being over time. This aligns with previous evidence, which pointed out an association between sleep difficulties and impaired psychological, social, and interpersonal functioning (Roberts et al., 2011). These results confirmed a deficiency of longitudinal studies considering the positive well-being aspects, compared to the studies evaluating mental health components in adolescents, yielding the need for more longitudinal research to enhance the role of positive components of health.

### Unlocking the teenage rhythm: exploring the association between chronotype and well-being

The phase of adolescents is delayed due to biological and physical changes, psychosocial interactions, active social life, early school hours, and inadequate sleep habits leading to a shift in eveningness. Despite that, few included studies evaluated the relation between chronotype at one time and mental health and well-being at a later time, showing that morning chronotype was associated with better mental health and positive well-being outcomes. From these results, it emerges that the bidirectional relation between sleep, mental health, and positive well-being should be considered a virtuous circle, and therefore, chronotype can play a crucial role in this cycle that should be further evaluated in future longitudinal research (Chaput et al., 2016).

### Limitations and suggestions for future research

The results of this systematic review should be considered in light of some limitations. First, most studies included in this review relied on self-report measures to assess sleep, and only a small percentage used actigraphy to evaluate sleep objectively. Since self-report data can be biased by social desirability and perception bias, evaluating sleep-related variables integrating subjective and objective methods is recommended to improve future research. To this end, future studies should, on the one hand, further explore the nature of this association using actigraphy that can provide a non-intrusive, advantageous way to estimate adolescents’ sleep objectively (Meltzer et al., 2012). On the other hand, more evidence is needed investigating sleep in adolescents using polysomnography (i.e., the current gold standard for measuring sleep, which measures physiologic parameters of sleep), which can provide a specific detection of distinct sleep architecture, sleep disturbances, and sleep parameters (Marino et al., 2013).

Second, studies evaluating positive well-being were limited and heterogeneous, often examining the relation between the two variables in a unidirectional way. This provides a scattered picture of the relation between sleep and health as conceptualized by the WHO and comprising both psychopathology and positive facets of well-being. Therefore, future longitudinal research is needed to disentangle how different aspects of sleep are reciprocally linked to adolescents’ health, as considered multidimensional.

Third, most of the included studies (50.8%) had a design with two time points and the average time-lag across studies was around one year (M = 14.2 months, SD = 9 months, ranging from 2 months to 3 years). Results showed that the time-lag between waves in assessing this relation is of utmost importance better to understand the underlying mechanisms of this association over time, as already documented for other psychosocial processes (e.g., Crocetti et al., 2021). For this reason, using at least three assessments to unravel the different bidirectional effects in the short- medium- and long-term is suggested.

Finally, concerning the implementation of research findings into clinical practice, a limitation of this meta-analysis is that it demonstrates a temporal virtuous circle between good sleep, low mental health issues, and high positive well-being, but does not allow for causal attributions. Poor sleep quality and lack of adequate sleep can contribute to the development of psychopathology, while good sleep quality and adequate sleep can promote positive well-being. These results suggest that addressing both sleep-related difficulties and mental health symptoms and positive well-being, rather than focusing on either alone, could be crucial in the clinical practice to effectively manage the cycle of sleep-mental health problems and positive well-being aspects.

## Funding

This research was supported by a grant from the 10.13039/100010663European Research Council (ERC) under the European Union's Horizon 2020 research and innovation programme (ERC-CoG IDENTITIES Grant agreement N. [101002163]; Principal investigator: Elisabetta Crocetti).

## Declaration of Competing Interest

The authors declare that they have no known competing financial interests or personal relationships that could have appeared to influence the work reported in this paper.

## Appendix Supplementary materials

Image, application 1
