# Supplementary material for: A meta-analysis of longitudinal studies on the interplay between sleep, mental health, and positive well-being in adolescents
Source: Int J Clin Health Psychol. 2023 Dec 2;24(1):100424. doi: 10.1016/j.ijchp.2023.100424 (PMC10730350; doi:10.1016/j.ijchp.2023.100424)

**Supplemental materials**

**Document S1: Prisma checklist**

| **Section and Topic** | **Item #** | **Checklist item** | **Location where item is reported** |
| --- | --- | --- | --- |
| **TITLE** | | |  |
| Title | 1 | Identify the report as a systematic review. | Title page |
| **ABSTRACT** | | |  |
| Abstract | 2 | See the PRISMA 2020 for Abstracts checklist. | Page 1 |
| **INTRODUCTION** | | |  |
| Rationale | 3 | Describe the rationale for the review in the context of existing knowledge. | Pages 2-6 |
| Objectives | 4 | Provide an explicit statement of the objective(s) or question(s) the review addresses. | Page 6 |
| **METHODS** | | |  |
| Eligibility criteria | 5 | Specify the inclusion and exclusion criteria for the review and how studies were grouped for the syntheses. | Page 7 |
| Information sources | 6 | Specify all databases, registers, websites, organisations, reference lists and other sources searched or consulted to identify studies. Specify the date when each source was last searched or consulted. | Page 7, Supplemental materials |
| Search strategy | 7 | Present the full search strategies for all databases, registers and websites, including any filters and limits used. | Page 7, Supplemental materials |
| Selection process | 8 | Specify the methods used to decide whether a study met the inclusion criteria of the review, including how many reviewers screened each record and each report retrieved, whether they worked independently, and if applicable, details of automation tools used in the process. | Page 7, Figure 2 |
| Data collection process | 9 | Specify the methods used to collect data from reports, including how many reviewers collected data from each report, whether they worked independently, any processes for obtaining or confirming data from study investigators, and if applicable, details of automation tools used in the process. | Page 7, Supplemental materials |
| Data items | 10a | List and define all outcomes for which data were sought. Specify whether all results that were compatible with each outcome domain in each study were sought (e.g. for all measures, time points, analyses), and if not, the methods used to decide which results to collect. | Page 7, Supplemental materials |
|  | 10b | List and define all other variables for which data were sought (e.g. participant and intervention characteristics, funding sources). Describe any assumptions made about any missing or unclear information. | Page 7, Supplemental materials |
| Study risk of bias assessment | 11 | Specify the methods used to assess risk of bias in the included studies, including details of the tool(s) used, how many reviewers assessed each study and whether they worked independently, and if applicable, details of automation tools used in the process. | n/a |
| Effect measures | 12 | Specify for each outcome the effect measure(s) (e.g. risk ratio, mean difference) used in the synthesis or presentation of results. | Page 8, Supplemental materials |
| Synthesis methods | 13a | Describe the processes used to decide which studies were eligible for each synthesis (e.g. tabulating the study intervention characteristics and comparing against the planned groups for each synthesis (item #5)). | Page 8, Supplemental materials |
|  | 13b | Describe any methods required to prepare the data for presentation or synthesis, such as handling of missing summary statistics, or data conversions. | Page 8, Supplemental materials |
|  | 13c | Describe any methods used to tabulate or visually display results of individual studies and syntheses. | Page 8, Supplemental materials |
|  | 13d | Describe any methods used to synthesize results and provide a rationale for the choice(s). If meta-analysis was performed, describe the model(s), method(s) to identify the presence and extent of statistical heterogeneity, and software package(s) used. | Page 8, Supplemental materials |
|  | 13e | Describe any methods used to explore possible causes of heterogeneity among study results (e.g. subgroup analysis, meta-regression). | Page 8, Supplemental materials |
|  | 13f | Describe any sensitivity analyses conducted to assess robustness of the synthesized results. | Page 8, Supplemental materials |
| Reporting bias assessment | 14 | Describe any methods used to assess risk of bias due to missing results in a synthesis (arising from reporting biases). | n/a |
| Certainty assessment | 15 | Describe any methods used to assess certainty (or confidence) in the body of evidence for an outcome. | Page 8, Supplemental materials |
| **RESULTS** | | |  |
| Study selection | 16a | Describe the results of the search and selection process, from the number of records identified in the search to the number of studies included in the review, ideally using a flow diagram. | Page 7, Supplemental materials |
|  | 16b | Cite studies that might appear to meet the inclusion criteria, but which were excluded, and explain why they were excluded. | Page 7, Supplemental materials, Figure 1 |
| Study characteristics | 17 | Cite each included study and present its characteristics. | Page 8, Supplemental materials |
| Risk of bias in studies | 18 | Present assessments of risk of bias for each included study. | n/a |
| Results of individual studies | 19 | For all outcomes, present, for each study: (a) summary statistics for each group (where appropriate) and (b) an effect estimate and its precision (e.g. confidence/credible interval), ideally using structured tables or plots. | Table 1 |
| Results of syntheses | 20a | For each synthesis, briefly summarise the characteristics and risk of bias among contributing studies. | Pages 8-12 |
|  | 20b | Present results of all statistical syntheses conducted. If meta-analysis was done, present for each the summary estimate and its precision (e.g. confidence/credible interval) and measures of statistical heterogeneity. If comparing groups, describe the direction of the effect. | Pages 8-12 |
|  | 20c | Present results of all investigations of possible causes of heterogeneity among study results. | Pages 8-12 |
|  | 20d | Present results of all sensitivity analyses conducted to assess the robustness of the synthesized results. | Pages 8-12 |
| Reporting biases | 21 | Present assessments of risk of bias due to missing results (arising from reporting biases) for each synthesis assessed. | n/a |
| Certainty of evidence | 22 | Present assessments of certainty (or confidence) in the body of evidence for each outcome assessed. | Pages 8-12 |
| **DISCUSSION** | | |  |
| Discussion | 23a | Provide a general interpretation of the results in the context of other evidence. | Pages 12-16 |
|  | 23b | Discuss any limitations of the evidence included in the review. | Pages 12-16 |
|  | 23c | Discuss any limitations of the review processes used. | Pages 15-16 |
|  | 23d | Discuss implications of the results for practice, policy, and future research. | Pages 15-16 |
| **OTHER INFORMATION** | | |  |
| Registration and protocol | 24a | Provide registration information for the review, including register name and registration number, or state that the review was not registered. | Page 6 |
|  | 24b | Indicate where the review protocol can be accessed, or state that a protocol was not prepared. | Page 6 |
|  | 24c | Describe and explain any amendments to information provided at registration or in the protocol. | n/a |
| Support | 25 | Describe sources of financial or non-financial support for the review, and the role of the funders or sponsors in the review. | Title page |
| Competing interests | 26 | Declare any competing interests of review authors. | Title page |
| Availability of data, code and other materials | 27 | Report which of the following are publicly available and where they can be found: template data collection forms; data extracted from included studies; data used for all analyses; analytic code; any other materials used in the review. | n/a |

*From:*  Page MJ, McKenzie JE, Bossuyt PM, Boutron I, Hoffmann TC, Mulrow CD, et al. The PRISMA 2020 statement: an updated guideline for reporting systematic reviews. BMJ 2021;372:n71. doi: 10.1136/bmj.n71

For more information, visit: <http://www.prisma-statement.org/>

**Document S2: Full search strategy**

The complete list of databases with related query strings and of the websites of the fifteen journals most likely to publish studies on the topic are reported below:

PubMed and MEDLINE

(((Sleep*[Title/Abstract] OR insomnia[Title/Abstract] OR polysomnogra*[Title/Abstract] OR REM[Title/Abstract] OR actigraph*[Title/Abstract] OR EEG [Title/Abstract] OR motor activity [Title/Abstract] OR circadian*[Title/Abstract] OR chronotype[Title/Abstract]) AND (pediatr*[Title/Abstract] OR paediatr*[Title/Abstract] OR teen*[Title/Abstract] OR school*[Title/Abstract] OR adolescen*[Title/Abstract] OR youth*[Title/Abstract] OR young*[Title/Abstract] OR child*[Title/Abstract])) AND (longitudinal*[Title/Abstract] OR prospective*[Title/Abstract] OR follow-up[Title/Abstract] OR daily[Title/Abstract] OR day-to-day[Title/Abstract] OR wave[Title/Abstract])))

EBSCO [PsycINFO, PsycArticles, ERIC]

AB (Sleep* OR insomnia OR polysomnogra* OR REM OR actigraph* OR EEG OR motor activity OR circadian* OR chronotype*) AND AB ( pediatr* OR paediatr* OR teen* OR school* OR adolescen* OR youth* OR young* OR child* ) AND AB ( longitudinal* OR prospective* OR follow-up OR daily OR day-to-day OR wave)

Web of Science

AB = (Sleep* OR insomnia OR polysomnogra* OR REM OR actigraph* OR EEG OR motor activity OR circadian* OR chronotype*) AND AB=(pediatr* OR paediatr* OR teen* OR school* OR adolescen* OR youth* OR young* OR child*) AND AB=(longitudinal* OR prospective* OR follow-up OR daily OR day-to-day OR wave)

ProQuest Dissertations and Theses

Ab (Sleep* OR insomnia OR polysomnogra* OR REM OR actigraph* OR EEG OR motor activity OR circadian* OR chronotype*) AND ab (pediatr* OR paediatr* OR teen* OR school* OR adolescen* OR youth* OR young* OR child*) AND ab(longitudinal* OR prospective* OR follow-up OR daily OR day-to-day OR wave)

Scopus

(ABS (sleep*  OR  insomnia  OR  polysomnogra*  OR  rem  OR  actigraph*  OR  eeg  OR  motor  AND activity  OR  circadian*  OR  chronotype*)  AND  ABS (pediatr*  OR  paediatr*  OR  teen*  OR  school*  OR  adolescen*  OR  youth*  OR  young*  OR  child*)  AND  ABS (longitudinal*  OR  prospective*  OR  follow-up  OR  daily  OR  day-to-day  OR  wave))

GreyNet

(Sleep* OR insomnia OR polysomnogra* OR REM OR actigraph* OR EEG OR motor activity OR circadian* OR chronotype*) AND (pediatr* OR paediatr* OR teen* OR school* OR adolescen* OR youth* OR young* OR child*) AND (longitudinal* OR prospective* OR follow-up OR daily OR day-to-day OR wave)

The screened journals were (in alphabetical order):

*BMC Public Health; Brain Development; Developmental Medicine and Child Neurology; Epilepsia; Epilepsy Behavior; International Journal of Environmental Research and Public Health; International Journal of Pediatrics Otorhinolaryntology; Journal of Child Neurology; Journal of Clinical Sleep Medicine*; *Journal of Sleep Research; Pediatrics; Plos One; Seizure European Journal of Epilepsy;* *Sleep; Sleep Medicine.*

Moreover, conference proceedings from sleep-related journals and reference lists of relevant published systematic reviews and meta-analyses were examined (see below for the full list). Finally, the reference lists of the included full-texts were screened to identify additional relevant studies.

**Full list of most relevant published systematic reviews and meta-analyses of which the reference lists were screened**

Beisbier, S., & Laverdure, P. (2020). Occupation-and activity-based interventions to improve performance of instrumental activities of daily living and rest and sleep for children and youth ages 5–21: A systematic review. *The American Journal of Occupational Therapy*, *74*(2), 7402180040p1-7402180040p32. <https://doi.org/10.5014/ajot.2020.039636>

Belmon, L. S., van Stralen, M. M., Busch, V., Harmsen, I. A., & Chinapaw, M. J. (2019). What are the determinants of children's sleep behavior? A systematic review of longitudinal studies. *Sleep Medicine Reviews*, *43*, 60-70. <https://doi.org/10.1016/j.smrv.2018.09.007>

Costa, S., Benjamin-Neelon, S. E., Winpenny, E., Phillips, V., & Adams, J. (2019). Relationship between early childhood non-parental childcare and diet, physical activity, sedentary behaviour, and sleep: A systematic review of longitudinal studies. *International Journal of Environmental Research and Public Health*, *16*(23), 4652. <https://doi.org/10.3390/ijerph16234652>

Ehsan, Z., Ishman, S. L., Kimball, T. R., Zhang, N., Zou, Y., & Amin, R. S. (2017). Longitudinal cardiovascular outcomes of sleep disordered breathing in children: A meta-analysis and systematic review. *Sleep*, *40*(3), zsx015. <https://doi.org/10.1093/sleep/zsx015>

Fatima, Y., Doi, S. A. R., & Mamun, A. A. (2015). Longitudinal impact of sleep on overweight and obesity in children and adolescents: A systematic review and bias‐adjusted meta‐analysis. *Obesity Reviews*, *16*(2), 137-149. <https://doi.org/10.1111/obr.12245>

Gronski, M., & Doherty, M. (2020). Interventions within the scope of occupational therapy practice to improve activities of daily living, rest, and sleep for children ages 0–5 years and their families: A systematic review. *The American Journal of Occupational Therapy*, *74*(2), 7402180010p1-7402180010p33. <https://doi.org/10.5014/ajot.2020.039545>

Guo, Y., Miller, M. A., & Cappuccio, F. P. (2021). Short duration of sleep and incidence of overweight or obesity in Chinese children and adolescents: A systematic review and meta-analysis of prospective studies. *Nutrition, Metabolism and Cardiovascular Diseases*, *31*(2), 363-371. <https://doi.org/10.1016/j.numecd.2020.11.001>

Li, L., Zhang, S., Huang, Y., & Chen, K. (2017). Sleep duration and obesity in children: A systematic review and meta‐analysis of prospective cohort studies. *Journal of Paediatrics and Child Health*, *53*(4), 378-385. <https://doi.org/10.1111/jpc.13434>

Miller, M. A., Kruisbrink, M., Wallace, J., Ji, C., & Cappuccio, F. P. (2018). Sleep duration and incidence of obesity in infants, children, and adolescents: A systematic review and meta-analysis of prospective studies. *Sleep*, *41*(4), zsy018. <https://doi.org/10.1093/sleep/zsy018>

Miller, M. A., Kruisbrink, M., Wallace, J., O’Keeffe, A., Valint, S., Ji, C., & Cappuccio, F. P. (2017). Abstract MP090: Sleep Duration Predict Incident Obesity in Childhood and Adolescence: Meta-analysis of Prospective Studies. *Circulation*, 135(suppl_1), AMP090. <https://doi/10.1161/circ.135.suppl_1.mp090>

Ruan, H., Xun, P., Cai, W., He, K., & Tang, Q. (2015). Habitual sleep duration and risk of childhood obesity: Systematic review and dose-response meta-analysis of prospective cohort studies. *Scientific Reports*, *5*(1), 1-14. <https://doi.org/10.1038/srep16160>

Scherrer, V., & Preckel, F. (2021). Circadian preference and academic achievement in school-aged students: A systematic review and a longitudinal investigation of reciprocal relations. *Chronobiology International*, *38*(8), 1195–1214. <https://doi.org/10.1080/07420528.2021.1921788>

Wu, Y., Gong, Q., Zou, Z., Li, H., & Zhang, X. (2017). Short sleep duration and obesity among children: A systematic review and meta-analysis of prospective studies. *Obesity Research & Clinical Practice*, *11*(2), 140-150. <https://doi.org/10.1016/j.orcp.2016.05.005>

**Document S3: Full coding procedure and protocol**

In the first section of the protocol, the following characteristics of the publication were coded: type of publication (i.e., journal article or grey literature), year of publication, and language of publication. The next section was related to the studies’ characteristics and included: funding sources (i.e., international, national funding, local funding, multiple funding sources); the number of waves of the longitudinal design; the time lag between waves; the dimensions of each study (coded according to the variables presented in Figure 1); and the method of assessment used to evaluate them (i.e., self-reports, objective assessment). In the third part, the following participants’ characteristics were extracted: sample size, gender composition of the sample (% females), mean age, geographical location, and ethnic composition of the sample.

Due to the high heterogeneity of the studies included, different effect sizes were extracted (i.e., odds ratio, hazard ratio, Pearson's correlation, Spearman's Rho correlation, and beta coefficient) to address how sleep variables, internalizing and externalizing problems, subjective and psychological well-being were longitudinally related (see Strategy of Analysis section). All the study authors of included studies for which data for effect size computations were not fully reported were contacted by e-mail to request missing or additional data. Specifically, 88 authors were contacted by e-mail (scheduling one reminder after two weeks if the authors did not answer the first request). Nine authors replied by providing the requested data; nine replied that they could not provide the required data (e.g., they could not access the dataset anymore); and 70 did not respond. The total number of studies included in the review accounts for 74 excluded because of insufficient data, as indicated in the PRISMA diagram (Figure 2).

**Document S4: Full strategy of analysis**

Using meta-analytic techniques, Pearson's correlations were computed to compare the effects across studies and calculate overall summary statistics. The random-effect model was used as a conservative approach to account for different sources of variation among studies (i.e., within-study and between-studies variance; Borenstein et al., 2010). Pearson's correlations were converted into Fisher's Z-scores for computational purposes and converted back into correlations for presentation Lipsey & Wilson, 2000). Correlations of |.10| were considered small, |.30| moderate, and |.50| large effect sizes respectively (Cohen, 1988; Ellis, 2010). Variance, standard error, 95% confidence interval, and statistical significance for each effect size were computed. Heterogeneity across studies was assessed with the *Q* statistic to test if it was statistically significant. Additionally, the *I*^2^ was used with values of 25%, indicating low levels of heterogeneity, and 50% and 75% indicating a moderate and high proportion of dispersion in the observed effects that would remain should sampling error be removed (Higgins et al., 2003). Moderator analyses were used to test which factors can account for the heterogeneity (Viechtbauer, 2007). Different moderators were tested using subgroup analysis (for categorical moderators, such as the method used to assess sleep) and meta-regression (for numerical moderators, such as the age of participants and time-lag between waves) when at least three studies for each moderator level were available (Crocetti, 2016). Finally, to account for potential publication bias, the visualization of the funnel plot was used (i.e., a scatter plot of the effect sizes estimated from individual studies against a measure of their precision, such as their standard errors). Without bias, the plot would be shaped as a symmetrical inverted funnel. However, since smaller or non-significant studies are less likely to be published, studies in the bottom left-hand corner of the plot are often omitted. Moreover, to evaluate the funnel plot, Egger's regression method (Egger et al., 1997) was also used, which statistically tests the asymmetry of the funnel plot, with non-significant results indicative of the absence of publication bias.

*References*

Borenstein, M., Hedges, L. V., Higgins, J. P. T., & Rothstein, H. R. (2010). A basic introduction to fixed-effect and random-effects models for meta-analysis. *Research Synthesis Methods*, *1*(2), 97–111. <https://doi.org/10.1002/jrsm.12>

Lipsey, M. W., & Wilson, D. B. (2000). *Practical meta-analysis* (Vol. 49). Sage Publications, Inc.

Cohen, J. (1988). Tha analysis of variance. In *Statistical power analysis for the behavioral sciences* (2nd ed., pagg. 273–406). Lawrence Erlbaum Associates.

Ellis, P. D. (2010). *The essential guide to effect sizes: Statistical power, meta-analysis, and the interpretation of research results*. Cambridge university press.

Higgins, J. P. T., Thompson, S. G., Deeks, J. J., & Altman, D. G. (2003). Measuring inconsistency in meta-analyses. *British Medical Journal*, *327*(7414), 557–560. <https://doi.org/10.1136/bmj.327.7414.557>

Viechtbauer, W. (2007). Accounting for heterogeneity via random-effects models and moderator analyses in meta-analysis. *Journal of Psychology*, *215*(2), 104–121. <https://doi.org/10.1027/0044-3409.215.2.104>

Crocetti, E. (2016). Systematic reviews with meta-analysis: Why, when, and how? *Emerging Adulthood*, *4*(1), 3–18. <https://doi.org/10.1177/2167696815617076>

Egger, M., Smith, G. D., Schneider, M., & Minder, C. (1997). Bias in meta-analysis detected by a simple, graphical test. *British Medical Journal*, *315*(7109), 629–634. <https://doi.org/10.1136/bmj.315.7109.629>

**Document S5: Study Characteristics**

Sixty-three studies were included in the systematic review. The main characteristics of the included studies are reported in Table 1. Regarding the characteristics of the publication, all studies, except for one that was a dissertation (Fairborn et al., 2010), were articles published in peer-reviewed journals. Around half of the included studies (53.9%) were published recently after 2019, and the remaining were published between 2018 and 2009 (46.1%). With regards to the study design, four studies used a daily design, and the remaining included two (50.8%), three (28.6%), or more than three (14.3%) time points. The average time lag between adjacent waves was around one year (M = 14.2 months, SD = 9 months, ranging from 2 months to 3 years). Most of the studies assessed sleep variables using self-report measures (93.3%), and the remaining studies (7.9%) used objective measures (i.e., actigraphy). Most studies (84.1%) reported one or multiple funding sources. The total number of participants at baseline was 130,500 (M = 2139, SD = 2752). Most samples were gender-balanced (the average percentage of females across samples was 53.3%; range 38.6%-76%), and the average age of sample participants at baseline was 14.2 years (SD = 1.9, range: 10-18 years). With regards to the geographic context, most of the included studies were conducted in the US (41.2%), Europe (20.6%), or China (17.5%). The remaining were conducted in Australia (6.3%), Canada (4.8%), Japan (4.8%), Brazil (1.6%), UK (1.6%), and Israel (1.6%)

| **Table 1:** Studies characteristics | | | | | | | | | | | | | | |
| --- | --- | --- | --- | --- | --- | --- | --- | --- | --- | --- | --- | --- | --- | --- |
| Authors and year | Funding | *N* waves | Time lag | Sleep dimension | Sleep assessment | Mental health and positive well-being variable | Mental health and positive well-being categorization | *N* participants baseline | *N* participants follow-up | % females | Mean age at baseline (in years) | Country | % ethnicity | |
|  |  |  |  |  |  |  |  |  |  |  |  |  |  |  |
| Alvaro et al., 2017 | n/a | 2 | 5-7 months | Symptoms of insomnia; Chronotype | Subjective measure (questionnaire) | Depression symptoms | Internalizing symptoms | 318 | 255 | 45.1% | 14.9 | Australia | n/a | |
| Ames et al, 2016 | Yes | 6 | 2 years | Sleep duration | Subjective measure (questionnaire) | General internalizing symptoms | Internalizing symptoms | 662 | 477 | 51.7% | 15.5 | Canada | 85% Caucasian | |
| Arnison et al., 2021 | Yes | 5 | 1 year | Symptoms of insomnia | Subjective measure (questionnaire) | Depression symptoms; Anxiety symptoms;  Positive affect | Internalizing symptoms; subjective well-being | 2767 | 1132 | 47.6% | 13.6 | Sweden | 24% immigrant background | |
| Bao-peng et al., 2020 | Yes | 3 | 1 year | Sleep duration | Subjective measure (questionnaire) | Depression symptoms | Internalizing symptoms | 11,83 | 4853 | 49.7% | 14.6 | China | n/a | |
| Barlett et al., 2012 | n/a | 3 | W1-W2: 7 months; W2- W3: 6 months | Sleep duration | Subjective measure (questionnaire) | Attention problems | Externalizing symptoms | 1196 | 1110 | 53% | 9.6 | USA | 90% white | |
| Bauducco et al., 2019 | Yes | 3 | 1 year | Sleep duration | Subjective measure (questionnaire) | Impulsive behavior | Externalizing symptoms | 2767 | 1982 | 47.6% | 13.7 | Sweden | 88.5% born in Sweden | |
| Bilsky et al., 2021 | Yes | 2 | 1 year | Symptoms of insomnia | Subjective measurement (questionnaire) | Anxiety symptoms | Internalizing symptoms | 2432 | 2432 | n/a | 14.9 | USA | n/a | |
| Catrett et al., 2009 | Yes | 2 | 1 Year | Symptoms of insomnia | Subjective measure (questionnaire) | Depression symptoms | Internalizing symptoms | 4353 | 4353 | 56% | 14.4 | USA | 11% Hispanic; 24% Black; 5% Asian; 7% American Indian; 9% other | |
| Conklin et al., 2018 | No | 3 | 6 months | Sleep duration | Subjective measure (questionnaire) | Depression symptoms | Internalizing symptoms | 3170 | 3071 | 53% | 14.8 | Canada | n/a | |
| Doane et al., 2015 | Yes | 3 | 6 months | Sleep duration; sleep quality (sleep efficiency);insomnia symptoms | Objective and subjective measures (actigraphy; questionnaire) | Depression symptoms; Anxiety symptoms | Internalizing symptoms | 82 | 71 | 76% | 18.1 | USA | 54% European American; 23% Latino/Hispanic descent; 13% Multi-racial; 5% African-American; 5% Asian American/Pacific Islander | |
| Erreygers et al., 2019 | Yes | 2 | 5 months | Sleep quality | Subjective measure (questionnaire) | Negative affect (Anger); Cyberbullying perpetratrion | Subjective well-being; Externalizing symptoms | 1748 | 1590 | 55.7% | 13.5 | Belgium | n/a | |
| Fairborn, 2010 | No | 2 | 1 year | Sleep duration | Subjective measure (questionnaire) | Depression symptoms; Self regulaiton | Internalizing symptoms; Psychological well-being | 14723 | 14723 | 51% | n/a | USA | 51.1% White; 21.5% African American; 16.5% Hispanic; 6.8% Asian; 2.6% American Indian; 1.5% reported no ethnic identity | |
| Falch et al., 2021 | Yes | 3 | 2 years | Symptoms of insomnia | Subjective measurement (questionnaire) | Emotional regulation | Psychological well-being | 1037 | 1037 | 49.5% | 12.50 | Norway | Ethnic origin of bio mother: 92.4% Norwegian; 3.3% Western country; 4.3% Other country. Ethnic origin of bio father: 90.5% Norwegian; 3.3% Western country; 4.3% Other country | |
| Frederiksen et al., 2014 | Yes | 3 | 1 year | Sleep duration | Subjective measure (questionnaire) | Depression symptoms; Self-esteem | Internalizing symptoms; Psychological well-being | 2256 | 2256 | 49.6% | n/a | USA | 82.2% European American; 6.9% Hispanic; 5.4% African American; 3.4% Asian American; 1.8% Multiracial; 0.3% Native American | |
| Genta et al., 2021 | n/a | 2 | 15 months | Sleep duration; Chronotype | Subjective measure (questionnaire) | Life satisfaction | Subjective well-being | 193 | 94 | 64% | 15 | Brazil | n/a | |
| Guo et al., 2021 | Yes | 2 | 2 years | Sleep duration | Subjective measurement (questionnaire) | Suicidal behavior | Internalizing symptoms | 3273 | 3145 | 51.1% | 13.7 | China | n/a | |
| Hayley et al., 2015 | Yes | 4 | T1-T2: 1 year; T2-T3: 1 year; T3-T4: 1 year | Symptoms of insomnia | Subjective measure (questionnaire) | Depression symptoms | Internalizing symptoms | 924 | 536 | 45.3% | n/a | Norway | n/a | |
| Haraden et al., 2017 | Yes | 4 | 3 years | Chronotype | Subjective measure (questionnaire) | Depression symptoms | Internalizing symptoms | 225 | 185 | 56.5% | 12.4 | USA | 82% White; 5% African American; 4% Asian; 1% American Indian; 8% Multiracial | |
| Itani et al., 2018 | Yes | 2 | 2 years | Sleep duration | Subjective measure (questionnaire) | Mental health status | Internalizing symptoms | 5687 | 3473 | 64% | n/a | Japan | n/a | |
| Jiang et al., 2022 | No | 2 | 5 months | Symptoms of insomnia | Subjective measurement (questionnaire) | Resilience | Psychological well-being | 188 | 188 | 48.2% | 13.8 | China | n/a | |
| Kalak et al., 2014 | Yes | 3 | 6 months | Sleep duration | Subjective measure (questionnaire) | Subjective psychological well-being | Psychological well-being | 886 | 886 | 51.8% | 13.3 | Switzerland | 55.34% German-speaking Swiss; 44.66% Norwegian | |
| Kaneita et al., 2009 | Yes | 2 | 2 years | Symptoms of insomnia | Subjective measure (questionnaire) | Mental health status | Internalizing symptoms | 681 | 516 | 43% | 13 | Japan | n/a | |
| Kechter & Leventhal, 2019 | Yes | 2 | 1 year | Symptoms of insomnia | Subjective measure (questionnaire) | Depressive symptoms | Internalizing symptoms | 3383 | 2309 | 56.1% | 15.5 | USA | 0.9% American Indian/Alaska Native; 20.4% Asian; 4.0% Black; 46.5% Hispanic; 4.5% Native Hawaiian/Pacific Islander; 5.9% Other; 1.3% can't choose | |
| Kenny et al., 2016 | Yes | Daily | Daily | Sleep duration | Subjective measure (sleep diary) | Negative affect (Sadness) | Subjective well-being | 208 | 208 | 64% | 15.9 | Ireland | 97% White; 0.5% Asian; 1.5% Black or Other | |
| Kortesoja et al., 2020 | Yes | 3 | 2 years | Sleep duration | Subjective measure (questionnaire) | Emotional difficulties; attention problems; conduct problems | Internalizing symptoms; externalizing symptoms | 8834 | 3712 | 51.1% | 13 | Finland | n/a | |
| Kuo et al., 2015 | Yes | 2 | 2 years | Sleep duration | Subjective measure (interview) | Depression symptoms; Risky behaviors risky behavior | Internalizing symptoms; externalizing symptoms | 246 | n/a | 51% | 17.7 | USA | 100% Mexian American | |
| Latina et al., 2021 | Yes | 3 | 1 year | Symptoms of insomnia | Subjective assessment (questionnaire) | Depression symptoms; self-harm | Internalizing symptoms | 1457 | 1339 | 47.3% | 13.2 | Sweden | | 89.2% born in Sweden |
| Leonard et al., 2021 | No | 2 | 6 months | Sleep duration | Subjective assessment (questionnaire) | Attention control difficulties | Externalizing symptoms | 345 | 319 | 47% | 12.6 | USA | 58.84% Non-Hispanic White/European; 29.28% Hispanic; 9.57% Non-Hispanic multiracial; 2.32% Non-Hispanic other (American Indian/Alaska Native, Asian, Black/African American) | |
| Lepore & Kliewer, 2013 | Yes | 2 | 6 months | Symptoms of insomnia | Subjective assessment (questionnaire) | Depressive symptoms | Internalizing symptoms | 498 | 498 | 56% | 12.8 | USA | 43% White/Caucasian; 24% Latino/a; 24% Black/African American; 9% other race/ethnicity | |
| Li et al., 2021 | Yes | 3 | 3 months | Insomnia symptoms; sleep duration; chronotype | Subjective assessment (questionnaire) | Depression symptoms; Anxiety symptoms | Internalizing symptoms | 1020 | 831 | 60.5% | 15.9 | China | n/a | |
| Lin et al., 2022 | Yes | 2 | 5 months | Sleep quality; sleep duration | Subjective assessment (questionnaire) | Depression; anxiety | Internalizing symptoms | 257 | 239 | 49% | 10.9 | China | n/a | |
| Liu et al., 2021 | Yes | 2 | 8 months | Sleep quality | Subjective assessment (questionnaire) | Cyberbullying perpetration; emotional distress | Externalizing symptoms; internalizing symptoms | 879 | 661 | 38.6% | 14.0 | China | n/a | |
| Liu et al., 2021 | Yes | 2 | 1 year | Daytime sleepiness | Subjective assessment (questionnaire) | Self-harm | Internalizing symptoms | 8629 | 7072 | 54.6% | 14.9 | China | n/a | |
| Liu et al., 2023 | Yes | 2 | 1 year | Insomnia symptoms | Subjective assessment (questionnaire) | Depression; Internet Gaming Disorder | Internalizing symptoms; externalizing symptoms | 7072 | 7072 | 50% | 14.6 | China | n/a | |
| Lovato et al., 2017 | n/a | 2 | 1 year | Sleep quality (sleep onset latency); sleep duration | Subjective assessment (questionnaire) | Depression symptoms | Internalizing symptoms | 345 | 138 | 40% | 15.7 | Australia | n/a | |
| Lundh et al., 2013 | Yes | 2 | 1 year | Insomnia symptoms | Subjective assessment (questionnaire) | Non-Suicidal Self-Injury | Internalizing symptoms | 992 | 881 | 51.1% | n/a | Sweden | n/a | |
| Luo et al., 2013 | Yes | 2 | 1 year | Insomnia symptoms | Subjective assessment (questionnaire) | Depression symptoms; Anxiety symptoms | Internalizing symptoms | 3763 | 2787 | 51.1% | 15 | China | n/a | |
| Marino et al., 2022 | Yes | 5 | 2 years | Insomnia symptoms | Subjective assessment (questionnaire) | Depression symptoms | Internalizing symptoms | 1113 | 1113 | 53.5% | 10 | Canada | n/a | |
| Matsumoto et al., 2021 | Yes | 2 | 2 years | Sleep quality | Subjective assessment (questionnaire) | Mental health status | Internalizing symptoms | 4836 | 3473 | 44.5% | n/a | Japan | n/a | |
| Maume, 2017 | Yes | 2 | 3 years | Sleep duration | Subjective assessment (questionnaire) | Depression symptoms | Internalizing symptoms | n/a | 974 | 50% | n/a | USA | 19% Non-white | |
| Nowakowski et al., 2016 | Yes | 3 | 1 year | Sleep duration | Subjective measure (questionnaire) | Depression symptoms | Internalizing symptoms | 1042 | 894 | 57% | 15.1 | USA | 31% Hispanic; 29% White; 28% African American; 8% Asian/Pacific Islander | |
| Roberts & Duong, 2013 | Yes | 2 | 1 year | Insomnia symptoms | Subjective measure (questionnaire) | Depression symptoms | Internalizing symptoms | 4179 | 3134 | 49.2% | n/a | USA | 37.01% European American; 34.59% African American; 23.64% Latino American; 4.75% Other | |
| Roberts & Duong, 2014 | Yes | 2 | 1 year | Sleep duration | Subjective measure (questionnaire) | Depression symptoms | Internalizing symptoms | 4179 | 3134 | 49.2% | n/a | USA | 37.01% European American; 34.59% African American; 23.64% Latino American; 4.75% Other | |
| Roberts et al., 2002 | Yes | 2 | 1 year | Insomnia symptoms | Subjective measure (questionnaire) | Self-esteem | Psychological well-being | 4179 | 3134 | 49.2% | n/a | USA | 37.01% European American; 34.59% African American; 23.64% Latino American; 4.75% Other | |
| Rosen et al., 2021 | Yes | 2 | 6 months | Sleep duration | Subjective measure (questionnaire) | General internalizing symptoms; general externalizing symptoms | Internalizing symptoms; externalizing symptoms | 154 | 121 | 46% | 14.3 | USA | 66% White; 11% Black; 11% Asian; 8% Hispanic or Latino; 3% Other | |
| Scherrer & Preckel, 2021 | Yes | 2 | 2 years | Chronotype | Subjective measure (questionnaire) | Self-efficacy | Self-efficacy | 764 | 764 | 52.7% | 16.8 | Germany | 89,60% Native German speakers | |
| Sladek et al., 2019 | Yes | Daily | Daily | Sleep duration | Objective measure (actigraphy) | Depression symptoms | Internalizing symptoms | 209 | 209 | 64.4% | 18.1 | USA | 85.1% Mexican; 10.1% South or Central Caribbean; 5.3% Cuban; 4.3% Other Latin American, Hispanic or Caribbean Heritage | |
| Soffer-Dudek & Sadeh, 2013 | Yes | 3 | 1 year | Sleep quality (sleep efficiency) | Objective measure (actigraphy) | General internalizing symptoms; general externalizing symptoms | Internalizing symptoms; externalizing symptoms | 94 | 71 | 56.4% | 10.5 | Israel | n/a | |
| Thorburn-Winsor et al., 2022 | Yes | 3 | 2 years | Sleep duration;  Bedtime | Objective measure (actigraphy) | Depression symptoms | Internalizing symptoms | 688 | 614 | 56.4% | 14.5 | UK | 92.44% Non minority; 7.6% Minority | |
| Troxel et al., 2019 | Yes | 4 | 1 year | Sleep duration;  symptoms of insomnia | Subjective measure (questionnaire) | Mental health status; risky behaviors | Internalizing symptoms; externalizing symptoms | 1850 | 1850 | 57% | 16.21 | USA | 21.6% White; 44% Hispanic; 21.1% Asian; 2.5% Black; 10.9% Other race | |
| Tu & Cai, 2020 | Yes | 2 | 7.40 months | Insomnia symptoms | Subjective measure (questionnaire) | Depression symptoms | Internalizing symptoms | 100 | 89 | 49% | 11.0 | USA | 57-63% European American; 43-37% Racial and ethnic minorities | |
| Tu et al., 2019 | Yes | 2 | 10 months | Sleep quality | Subjective measure (questionnaire) | Depression symptoms; Anxiety symptoms | Internalizing symptoms | 123 | 99 | 50% | 12.0 | USA | European americans: 58.5%; African Americans: 35%; Other: 6.5% | |
| Vaszonyi et al., 2021 | n/a | 3 | 1 year | Sleep quality | Subjective measure (questionnaire) | General externalizing symptoms; depression symptoms; anxiety symptoms; self-esteem | Internalizing symptoms; externalizing symptoms; psychological well-being | 586 | 457 | 58.4% | 12.3 | Czech Republic | n/a | |
| Vaszonyi et al., 2022 | n/a | 4 | 6 months | Sleep quality | Subjective measure (questionnaire) | Depression and anxiety symptoms | Internalizing symptoms | 570 | 570 | 58.6% | 12.4 | Czech Republic | 97.8% Czech ethnicity | |
| Vernon et al., 2017 | Yes | 3 | 1 year | Insomnia symptoms | Subjective measure (questionnaire) | Externalizing behaviors; depression symptoms | Externalizing symptoms; internalizing symptoms | 874 | 874 | 59% | 14.4 | Australia | 57.2% Caucasian; 7.2% Asian; 1.6% Aboriginal or Torres Strait Islander; 23.3% Other; 10.6% did not respond | |
| Vernon et al., 2018 | Yes | 4 | 1 year | Sleep quality | Subjective measure (questionnaire) | Self-esteem | Psychological well-being | n/a | 1101 | 57% | 13.5 | Australia | 56.9% Caucasian; 7.1% Asian; 2% Aboriginal or Torres Strait Islander; 21.9% Other; 11.9% did not respond | |
| Wang et al., 2020 | Yes | 4 | 6 months | Sleep quality | Subjective measure (questionnaire) | Resilience | Psychological well-being | 1299 | 840 | 58% | 15.8 | China | n/a | |
| Wang & Yip, 2020 | Yes | Daily | Daily | Daily Sleep | Objective measure (actigraphy) | Negative affect; Positive affect; Life satisfaction | Subjective well-being | 256 | 256 | 73% | 14.7 | USA | 40% Asian; 22% Black; 38% Latinx | |
| Wong & Brower, 2012 | Yes | 3 | T1-T2: 1 Year; T2-T3: 5 years | Insomnia symptoms | Subjective measure (questionnaire) | Suicidal intention | Internalizing symptoms | 6504 | 6504 | n/a | 15.9 | USA | n/a | |
| Wong et al., 2015 | Yes | 3 | T1-T2: 1 Year; T2-T3: 5 years | Insomnia symptoms | Subjective measure (questionnaire) | Substance abuse | Externalizing symptoms | 6504 | 6504 | n/a | 15.9 | USA | n/a | |
| Yip et al., 2022 | Yes | Daily | Daily | Insomnia symptoms | Subjective measure | Anxiety symptoms; positive and negative affects | Internalizing symptoms; Subjective well-being | 350 | 350 | 69% | 14.3 | USA | 22.6% Black; 41.4% Asian; 36.9% Latino | |
| Zeiders, 2017 | Yes | 2 | 2 years | Sleep duration; sleep quality | Subjective measure (sleep diary) | General internalizing symptoms | Internalizing symptoms | 113 | 84 | 49.6% | 15.7 | USA | 85.8% born in the US | |
| Zhang et al., 2022 | Yes | 2 | 2 months | Sleep duration | Subjective assessment (questionnaire) | Mental health status | Internalizing symptoms | 2427 | 2427 | n/a | n/a | China | n/a | |
| *Notes:* All studies were journal articles except for Fairborn et al., 2010 | | | | | | | | | | | | | | |

*References*

Ames, M. E., Holfeld, B., & Leadbeater, B. J. (2016). Sex and age group differences in the associations between sleep duration and BMI from adolescence to young adulthood. *Psychology & Health*, *31* (8), 976–992. https://doi.org/10.1080/08870446.2016.1163360

Arnison, T., Schrooten, M., Hesser, H., Jansson-Fröjmark, M., & Persson, J. (2021). Longitudinal, bidirectional relationships of insomnia symptoms and musculoskeletal pain across adolescence: the mediating role of mood. *Pain*, *163* (2), 287–298. <https://doi.org/10.1097/j.pain.0000000000002334>

Bauducco, S., Flink, I., Boersma, K., & Linton, S. J. (2019). Preventing sleep deficit in adolescents: Long‐term effects of a quasi‐experimental school‐based intervention study. *Journal of Sleep Research*, *29*(1), e12940. <https://doi.org/10.1111/jsr.12940>

Bilsky, S. A., Luber, M. J., Cloutier, R. M., Dietch, J. R., Taylor, D. J., & Friedman, H. P. (2021). Cigarette use, anxiety, and insomnia from adolescence to early adulthood: A longitudinal indirect effects test. *Addictive Behaviors*, *120*, 106981. <https://doi.org/10.1016/j.addbeh.2021.106981>

Catrett, C. D., & Gaultney, J. F. (2009). Possible insomnia predicts some risky behaviors among adolescents when controlling for depressive symptoms. *Journal of Genetic Psychology*, *170*(4), 287–309. <https://doi.org/10.1080/00221320903218331>

Conklin, A., Yao, C. A., & Richardson, C. G. (2018). Chronic sleep deprivation and gender-specific risk of depression in adolescents: a prospective population-based study. *BMC Public Health*, *18*(1), 1–7. <https://doi.org/10.1186/s12889-018-5656-6>

Doane, L. D., & Thurston, E. C. (2013). Associations among sleep, daily experiences, and loneliness in adolescence: Evidence of moderating and bidirectional pathways. *Journal of Adolescence*, *37*(2), 145–154. <https://doi.org/10.1016/j.adolescence.2013.11.009>

Erreygers, S., Vandebosch, H., Vranjes, I., Baillien, E., & De Witte, H. (2018). The longitudinal association between poor sleep quality and cyberbullying, mediated by anger. *Health Communication*, *34*(5), 560–566. <https://doi.org/10.1080/10410236.2017.1422098>

Fairborn, S. K. (2010). *The effects of community violence exposure on adolescents' health*. University of California, Riverside.

Falch-Madsen, J., Wichstrøm, L., Pallesen, S., Ranum, B. M., & Steinsbekk, S. (2021). Child and family predictors of insomnia from early childhood to adolescence. *Sleep Medicine*, *87*, 220–226. <https://doi.org/10.1016/j.sleep.2021.08.023>

Fredriksen, K., Rhodes, J. E., Reddy, R., & Way, N. (2004). Sleepless in Chicago: Tracking the effects of adolescent sleep loss during the middle school years. *Child Development*, *75*(1), 84–95. <https://doi.org/10.1111/j.1467-8624.2004.00655.x>

Guo, L., Wang, W., Wang, T., Zhao, M., Wu, R., & Lu, C. (2020). The longitudinal association between sleep duration and suicidal behavior among Chinese adolescents: The role of nonmedical use of prescription drug. *Behavioral Sleep Medicine*, *19*(5), 589–601. <https://doi.org/10.1080/15402002.2020.1822361>

Hayley, A. C., Skogen, J. C., Sivertsen, B., Wold, B., Berk, M., Pasco, J. A., & Øverland, S. (2015). Symptoms of depression and difficulty initiating sleep from early adolescence to early adulthood: A longitudinal study. *Sleep*, *38*(10), 1599–1606. <https://doi.org/10.5665/sleep.5056>

Itani, O., Kaneita, Y., Doi, K., Tokiya, M., Jike, M., Nakagome, S., Otsuka, Y., Ohida, T. (2018). Longitudinal epidemiologic study of poor mental health status in Japanese adolescents: Incidence of predictive lifestyle factors. *The Journal of Clinical Psychiatry*, *79* (4), 15114.

Jakobsson, M., Sundin, K., Högberg, K., & Josefsson, K. (2020). “I want to sleep, but I can’t”: Adolescents’ lived experience of sleeping difficulties. *Journal of School Nursing*, *38* (5), 449–458. <https://doi.org/10.1177/1059840520966011>

Jiang, H., Yu, W., Lin, D., & Macnamara, B. N. (2021). Resilience of adolescents, though weakened during pandemic-related lockdown, serves as a protection against depression and sleep problems. *Psychology Health & Medicine*, *27* (9), 1977–1988. <https://doi.org/10.1080/13548506.2021.1990367>

Kalak, N., Lemola, S., Brand, S., Holsboer–Trachsler, E., & Grob, A. (2014). Sleep duration and subjective psychological well-being in adolescence: A longitudinal study in Switzerland and Norway. *Neuropsychiatric Disease and Treatment*, *10*, 1199-1207. https://doi.org/10.2147/NDT.S62533

Kaneita, Y., Yokoyama, E., Harano, S., Tamaki, T., Suzuki, H., Munezawa, T., Nakajima, H., Asai, T., & Ohida, T. (2009). Associations between sleep disturbance and mental health status: A longitudinal study of Japanese junior high school students. *Sleep Medicine*, *10*(7), 780–786. <https://doi.org/10.1016/j.sleep.2008.06.014>

Kechter, A., & Leventhal, A. M. (2018). Longitudinal association of sleep problems and distress tolerance during adolescence. *Behavioral Medicine*, *45*(3), 240–248. <https://doi.org/10.1080/08964289.2018.1514362>

Kuo, S. I., Updegraff, K. A., Zeiders, K. H., McHale, S. M., Umaña-Taylor, A. J., & De Jesús, S. A. R. (2014). Mexican American adolescents’ sleep patterns: Contextual correlates and implications for health and adjustment in young adulthood. *Journal of Youth and Adolescence*, *44* (2), 346–361. <https://doi.org/10.1007/s10964-014-0156-1>

Latina, D., Bauducco, S., & Tilton-Weaver, L. (2020). Insomnia symptoms and non‐suicidal self‐injury in adolescence: Understanding temporal relations and mechanisms. *Journal of Sleep Research*, *30* (1), e13190. <https://doi.org/10.1111/jsr.13190>

Leonard, H., & Khurana, A. (2022). Parenting behaviors and family conflict as predictors of adolescent sleep and bedtime media use. *Journal of Youth and Adolescence*, *51*(8), 1611–1621. <https://doi.org/10.1007/s10964-022-01614-4>

Lepore, S. J., & Kliewer, W. (2013). Violence exposure, sleep disturbance, and poor academic performance in middle school. *Journal of Abnormal Child pPsychology*, *41*, 1179-1189. https://doi.org/10.1007/s10802-013-9709-0

Li, Y., Zhou, Y., Ru, T., Niu, J., He, M., & Zhou, G. (2021b). How does the COVID-19 affect mental health and sleep among Chinese adolescents: a longitudinal follow-up study. *Sleep Medicine*, *85*, 246–258. <https://doi.org/10.1016/j.sleep.2021.07.008>

Lin, C. Y., Potenza, M. N., Ulander, M., Broström, A., Ohayon, M. M., Chattu, V. K., & Pakpour, A. H. (2021). Longitudinal relationships between nomophobia, addictive use of social media, and insomnia in adolescents. *Healthcare*, *9* (9), 1201. <https://doi.org/10.3390/healthcare9091201>

Liu, B., Wang, X., Liu, Z., Wang, Z., An, D., Wei, Y., Jia, C., & Liu, X. (2020b). Depressive symptoms are associated with short and long sleep duration: A longitudinal study of Chinese adolescents. *Journal of Affective Disorders*, *263*, 267–273. <https://doi.org/10.1016/j.jad.2019.11.113>

Liu, C., Liu, Z., & Yuan, G. (2020a). Associations between cyberbullying perpetration, sleep quality, and emotional distress among adolescents. *Journal of Nervous and Mental Disease*, *209* (2), 123–127. <https://doi.org/10.1097/nmd.0000000000001267>

Lovato, N., Short, M. A., Micic, G., Hiller, R., & Gradisar, M. (2017). An investigation of the longitudinal relationship between sleep and depressed mood in developing teens. *Nature and Science of Sleep*, *9*, 3–10. <https://doi.org/10.2147/nss.s111521>

Lundh, L., Bjärehed, J., & Wångby-Lundh, M. (2012). Poor sleep as a risk factor for nonsuicidal Self-Injury in adolescent girls. *Journal of Psychopathology and Behavioral Assessment*, *35* (1), 85–92. <https://doi.org/10.1007/s10862-012-9307-4>

Luo, C., Zhang, J., & Pan, J. (2013). One-Year course and effects of insomnia in rural Chinese adolescents. *Sleep*, *36*(3), 377–384. <https://doi.org/10.5665/sleep.2454>

Marino, C., Andrade, B. F., Montplaisir, J., Petit, D., Touchette, E., Paradis, H., Côté, S. M., Tremblay, R. E., Szatmari, P., & Boivin, M. (2022). Testing bidirectional, longitudinal associations between disturbed sleep and depressive symptoms in children and adolescents using cross-lagged models. *Jama Network Open*, *5* (8), e2227119. <https://doi.org/10.1001/jamanetworkopen.2022.27119>

Matsumoto, Y., Kaneita, Y., Itani, O., Otsuka, Y., & Kinoshita, Y. (2021). Longitudinal epidemiological study of subjective sleep quality in Japanese adolescents to investigate predictors of poor sleep quality. *Sleep and Biological Rhythms*, *20* (1), 87–96. <https://doi.org/10.1007/s41105-021-00343-8>

Maume, D. J. (2017). Social relationships and the sleep-health nexus in adolescence: Evidence from a comprehensive model with bi-directional effects. *Sleep Health*, *3* (4), 284–289. <https://doi.org/10.1016/j.sleh.2017.05.006>

Nowakowski, S., Choi, H. J., Meers, J. M., & Temple, J. (2016). Inadequate sleep as a mediating variable between exposure to interparental violence and depression severity in adolescents. *Journal of Child & Adolescent Trauma*, *9*(2), 109–114. <https://doi.org/10.1007/s40653-016-0091-2>

Roberts, R. E., & Duong, H. T. (2013). Depression and insomnia among adolescents: A prospective perspective. *Journal of Affective Disorders*, *148* (1), 66–71. <https://doi.org/10.1016/j.jad.2012.11.049>

Roberts, R. E., & Duong, H. T. (2014). The prospective association between sleep deprivation and depression among adolescents. *Sleep*, *37*(2), 239–244. <https://doi.org/10.5665/sleep.3388>

Sladek, M. R., Doane, L. D., Gonzales, N. A., Grimm, K. J., & Luecken, L. J. (2019). Latino adolescents’ cultural values associated with diurnal cortisol activity. *Psychoneuroendocrinology*, *109*, 104403. <https://doi.org/10.1016/j.psyneuen.2019.104403>

Soffer-Dudek, N., & Sadeh, A. (2012). Dream recall frequency and unusual dream experiences in early adolescence: Longitudinal links to behavior problems. *Journal of Research on Adolescence*, *23* (4), 635–651. <https://doi.org/10.1111/jora.12007>

Thorburn-Winsor, E. A., Neufeld, S., Rowthorn, H., Van Sluijs, E. M. F., Brage, S., Jones, P. B., Goodyer, I., & Winpenny, E. (2022). Device-measured sleep onset and duration in the development of depressive symptoms in adolescence. *Journal of Affective Disorders*, *310*, 396–403. <https://doi.org/10.1016/j.jad.2022.05.051>

Troxel, W., Rodriguez, A., Seelam, R., Tucker, J. S., Shih, R. A., & D’Amico, E. J. (2019). Associations of longitudinal sleep trajectories with risky sexual behavior during late adolescence. *Health Psychology*, *38* (8), 716–726. <https://doi.org/10.1037/hea0000753>

Tu, K. M., & Cai, T. (2020). Reciprocal associations between adolescent peer relationships and sleep. *Sleep Health*, *6* (6), 743–748. <https://doi.org/10.1016/j.sleh.2020.01.019>

Tu, K. M., Spencer, C. W., El-Sheikh, M., & Erath, S. A. (2019). Peer victimization predicts sleep problems in early adolescence. *Journal of Early Adolescence*, *39* (1), 67–80. <https://doi.org/10.1177/0272431617725199>

Vazsonyi, A. T., Liu, D., & Blatný, M. (2022). Longitudinal bidirectional effects between sleep quality and internalizing problems. *Journal of Adolescence*, *94* (3), 448–461. <https://doi.org/10.1002/jad.12039>

Vazsonyi, A. T., Liu, D., Javakhishvili, M., Beier, J., & Blatný, M. (2021). Sleepless: The developmental significance of sleep quality and quantity among adolescents. *Developmental Psychology*, *57* (6), 1018–1024. <https://doi.org/10.1037/dev0001192>

Vernon, L., Modecki, K. L., & Barber, B. L. (2017). Tracking effects of problematic social networking on adolescent psychopathology: The mediating role of sleep disruptions. *Journal of Clinical Child & Adolescent Psychology*, *46* (2), 269-283. https://doi.org/10.1080/15374416.2016.1188702

Vernon, L., Modecki, K. L., & Barber, B. L. (2018). Mobile phones in the bedroom: Trajectories of sleep habits and subsequent adolescent psychosocial development. *Child Development*, *89* (1), 66-77. https://doi.org/10.1111/cdev.12836

Wang, J., Zhang, X., Simons, S., Sun, J., Shao, D., & Cao, F. (2020). Exploring the bi-directional relationship between sleep and resilience in adolescence. *Sleep Medicine*, *73*, 63–69. <https://doi.org/10.1016/j.sleep.2020.04.018>

Wang, Y., & Yip, T. (2019). Sleep facilitates coping: moderated mediation of daily sleep, Ethnic/Racial discrimination, stress responses, and adolescent well‐being. *Child Development*, *91*,4. <https://doi.org/10.1111/cdev.13324>

Wong, M. M., & Brower, K. J. (2012). The prospective relationship between sleep problems and suicidal behavior in the National Longitudinal Study of Adolescent Health. *Journal of Psychiatric Research*, *46* (7), 953–959. <https://doi.org/10.1016/j.jpsychires.2012.04.008>

Zeiders, K. H. (2017). Discrimination, daily stress, sleep, and Mexican-origin adolescents’ internalizing symptoms. *Cultural Diversity & Ethnic Minority Psychology*, *23* (4), 570–575. <https://doi.org/10.1037/cdp0000159>

Zhang, L., Yang, Y., Luo, Y., Liu, Z. Z., Jia, C. X., & Liu, X. (2022). A longitudinal study of insomnia, daytime sleepiness, and academic performance in Chinese adolescents. *Behavioral Sleep Medicine*, *20* (6), 798-808. https://doi.org/10.1080/15402002.2021.2021202

**Document S6: Effect Sizes of the Included Studies**

| **Study** | **Sleep variable (Method of assessment)** | **Mental health and positive well-being variable** | **Mental health and positive well-being category for meta-analytic calculation** | **Sleep variables T1 → Mental health and positive well-being variables T2^2^ (Effect size reported)** | **Sleep variables T1 → Mental health and positive well-being variables T2^2^ (Effect size expressed as Pearson’s correlations ^a^)** | **Mental health and positive well-being variables T1 → Sleep variables T2^1^ (Effect size reported)** | **Mental health and positive well-being variables T1 → Sleep variables T2^1^ (Effect size expressed as Pearson’s correlations ^b^)** | **Main findings** |
| --- | --- | --- | --- | --- | --- | --- | --- | --- |
| Alvaro et al., 2017 * | Symptoms of insomnia  (S)  Chronotype  (S) | Depression symptoms; anxiety symptoms | Internalizing symptoms | Symptoms of insomnia T1 – Depression T2:  *r* =.59^***^    Symptoms of insomnia T1 – Anxiety T2:  *r* = .41^***^  Morningness preference T1 – Depression T2:  *r* =-.30^*^  Morningness preference T1 – Anxiety T2:  *r* =-.15^*^ | Symptoms of insomnia T1 – Depression T2:  *r* = -.59^***^  [-.67, -.51]    Symptoms of insomnia T1 – Anxiety T2:  *r* = -.41^***^  [-.52, -.30]  Morningness preference T1 – Depression T2:  *r* =-.30^*^  [-.41, -.19]  Morningness preference T1 – Anxiety T2:  *r* =-.15^*^  [-.27, -.03] | Depression T1 – Symptoms of insomnia T2:  *r* =.67^***^    Anxiety T1 – Symptoms of insomnia T2:  *r* = .47^***^  Depression T1 – Morningness preference T2:  *r* =-.30^*^    Anxiety T1 – Morningness preference T2:  *r* = -.21^*^ | Depression:  *r* = -.67^***^  [-.74, -.60]    Anxiety:  *r* = -.47^***^  [-.57, -.37]  Depression T1 – Morningness preference T2:  *r* =-.32^*^  [-.43,-.21]    Anxiety T1 – Morningness preference T2:  *r* = -.21^*^  [-.33, -.09] | Higher symptoms of insomnia were bidirectionally associated with higher levels of depression and anxiety symptoms over time. Moreover, morningness preference was associated with lower depression and anxiety symptoms over time. |
| Ames et al, 2016 * | Sleep duration  (S) | Internalizing symptomatology | Internalizing symptoms | *r* = -.18^***^ | *r* = -.18^***^  [-.27, -.10] | *r* = -.04 | *r* = -.04  [-.13, .05] | Longer sleep duration was associated with lower internalizing symptoms over time but not vice versa. |
| Arnison et al., 2021 * | Symptoms of insomnia  (S) | Depression symptoms; anxiety symptoms;  Positive affect | Internalizing symptoms;  Subjective well-being | Depression:  *r* = .30^***^  Anxiety:  *r* = .32^***^  Positive affect:  *r* = .23^***^ | Depression:  *r* = -.30^***^  [-.36, -.25]  Anxiety:  *r* = -.32^***^  [-.38, -.27]  Positive affect:  *r* = .23^***^  [.18, .29] | Depression:  *r* =.27^***^  Anxiety  *r* = .28^***^ | Depression:  *r* = - .27^***^  [-.33, -.22]  Anxiety  *r* = -.28^***^  [-.34, -.23] | Higher symptoms of insomnia were bidirectionally linked with higher depression and anxiety symptoms over time.  Moreover, a higher level of insomnia was associated with higher positive affect over time. |
| Bao-peng et al., 2020 | Sleep duration  (S) | Depression symptoms | Internalizing symptoms | OR Short sleep duration on weekdays  (<6 hours vs. 8 hours):  4.95^**^  [3.92–6.24]  OR Short sleep duration weekend  (<6 hours vs. 8 hours):  2.14^**^  [1.76–2.61] |  |  |  | Short sleep duration (< 6 hours) both during weekends and weekdays was associated with a higher risk of internalizing symptoms over time. |
| Barlett et al., 2012 * | Sleep duration  (S) | Attention problems | Externalizing symptoms | *r* = -.16^***^ | *r* = -.16^***^  [-.22, -.16] | *r* = -.09^*^ | *r* = -.09^*^  [-.15, -.03] | Longer sleep duration was bidirectionally associated with lower externalizing symptoms over time. |
| Bauducco et al., 2019 * | Sleep duration  (S) | Antisocial behavior | Externalizing symptoms | *r* = -.20^***^ | *r* = -.20^***^  [-.25, -.16] | *r* = -.17^**^ | *r* = -.17^***^  [-.22, -.13] | Longer sleep duration was bidirectionally associated with lower externalizing symptoms over time. |
| Bilsky et al., 2021 * | Symptoms of insomnia  (S) | Anxiety symptoms | Internalizing symptoms | *r =* .27^***^ | *r* = -.27^***^  [-.31, -.24] | *r* = .13^***^ | *r* = -.13^***^  [-.14, -.11] | Higher symptoms of insomnia were bidirectionally associated with higher depression and anxiety symptoms over time. |
| Catrett et al., 2009 * | Symptoms of insomnia  (S) | Depression symptoms | Internalizing symptoms | Depression:  *ρ*: .24^***^ | Depression:  *r* = -.25^***^  [-.28, -.22] |  |  | Higher symptoms of insomnia were associated with higher levels of depression symptoms over time. |
| Conklin et al., 2018 | Sleep duration  (S) | Depression symptoms | Internalizing symptoms | n/a | n/a | n/a | n/a | Short sleep duration was associated with increased levels of depression symptoms in females but not in males. |
| Doane et al., 2015 * | Sleep duration; sleep quality (sleep efficiency); insomnia symptoms  (O) | Depression symptoms; anxiety symptoms | Internalizing symptoms | Sleep quality T1 – Depression T2: *r* = -.02  Sleep duration T1 – Depression T2:  *r* =.12  Sleep problems T1 – Depression T2: *r* =.29^*^  Sleep quality T1 – Anxiety T2:  *r* = -.02  Sleep duration T1 – Anxiety T2: *r* = .10  Sleep problems T1- Anxiety T2: *r* = .28^*^ | Sleep quality T1 – DepressionT2:  *r* = -.02  [-.26, -.22]  Sleep duration T1 – Depression T2:  *r* =.12  [-.28, .20]  Sleep problems T1 – Depression T2:  *r* = -.29^*^  [-.51, -.07]  Sleep quality T1 – Anxiety T2:  *r* = -.02  [-.26, .22]  Sleep duration T1 – Anxiety T2:  *r* = .10  [-.14, .34]  Sleep problems T1- Anxiety T2:  *r* = -.28^*^  [-.50, -.06] | Depression T1- Sleep quality T2: *r* = -.01  Depression T1 – Sleep duration T2:  *r* = .09  Depression T1 – Sleep problems T2:  *r* =.28^*^  Anxiety T1 – Sleep quality:  *r* = -.03  Anxiety T1 – Sleep duration:  *r* =.09  Anxiety T1 – Sleep problems:  *r* = .04 | Depression T1- Sleep quality T2:  *r* = -.01  [-.25, .23]  Depression T1 – Sleep duration T2:  *r* = .09  [-.15, .33]  Depression T1 – Sleep problems T2:  *r* =.28^*^  [.06, .50]  Anxiety T1 – Sleep quality:  *r* = -.03  [-.27, .21]  Anxiety T1 – Sleep duration:  *r* =.09  [-.15, .33]  Anxiety T1 – Sleep problems:  *r* = .04  [-.20, .28] | Sleep problems were bidirectionally associated with higher levels of depression over time. Moreover, sleep problems were linked to higher levels of anxiety symptoms over time, but not vice versa.  No associations were found between sleep quality, sleep duration, and depression and anxiety over time. |
| Erreygers et al., 2019 * | Sleep quality (S) | Negative affect (Anger); Cyberbullying perpetration | Subjective well-being; Externalizing symptoms | Negative affect:  *r* = -.29^***^  Bullying:  *r* = -.19^***^ | Negative affect:  *r* = .29^***^  [.25, .34]  Bullying:  *r* = -.19^***^  [-.25, -.16] |  |  | Higher sleep quality was associated with higher subjective well-being and lower externalizing symptoms over time. |
| Fairborn, 2010 * | Sleep duration; Sleep quality  (S) | Depressive symptoms; Self-regulation | Internalizing symptoms; Psychological well-being |  |  | Depression T1 – Sleep duration T2:  *r* = - .11^***^  Self Regulation T1- Sleep duration T2:  -.09^***^ | Depression T1 – Sleep duration T2:  *r* = - .11^***^  [.09, .13]  Self Regulation T1- Sleep duration T2:  -.09^***^  [-.11,-.07] | Higher levels of internalizing symptoms and psychological well-being were associated with shorter sleep duration over time. |
| Falch et al., 2021 * | Symptoms of insomnia  (S) | Self regulation | Psychological well-being | *r* = -.10^*^ | *r* = .10^*^  [.04, .16] | *r* = -.08 | *r* = -.08  [.02, .14] | Symptoms of insomnia were associated with lower psychological well-being over time but not vice versa. |
| Frederiksen et al., 2014 * | Sleep duration  (S) | Depression symptoms; Self-esteem | Internalizing symptoms; Psychological well-being | Sleep duration T1 – Depression symptoms T2:  *r* = -.18^***^  Sleep duration T1 – Self-esteem T2  *r* = .13 | Sleep duration T1 – Depression symptoms T2:  *r* = -.18^***^  [-.22, -.14]  Sleep duration T1 – Self-esteem T2  *r* = .13  [.09, .17] | Depression symptoms T1 –Sleep duration T2:  *r* = -.19^**^  Self-esteem T1 – Sleep duration T2  *r* = .14^**^ | Depression symptoms T1 – Sleep duration T2:  *r* = -.19^**^  [-.23, -.15]  Self-esteem T1 – Sleep duration T2  *r* = .14^**^  [.10, .18] | Longer sleep duration was bidirectionally associated with lower levels of depression symptoms over time.  Moreover, self-esteem was associated with longer sleep duration over time, but not vice versa. |
| Genta et al., 2021 * | Poor sleep quality  (S) | Life satisfaction | Subjective well-being | *ρ* = -0.39^***^ | *r* = .41^***^  [.24, .58] | *ρ* = -0.52^***^ | *r* = .54^***^  [.40, .69] | Lower levels of sleep quality were bidirectionally associated with lower levels of subjective well-being over time. |
| Guo et al., 2021 | Sleep duration  (S) | Suicidal ideation | Internalizing symptoms | Short sleep duration (<7 hours Vs. 7-9 hours of sleep) OR: 1.96^***^  [1.59 – 2.41] |  |  |  | Short sleep duration (< 7 hours) was associated with a higher risk of internalizing symptoms over time. |
| Hayley et al., 2015 | Symptoms of insomnia  (S) | Depression symptoms | Internalizing symptoms | T1–T2:  *r* = .19^***^;  T2–T3:  *r* = .22^***^;  T3–T4:  *r* = .27^***^ |  |  |  | Associations between insomnia symptoms and depression were comparably high across time. |
| Haraden et al., 2017 | Circadian preference  (S) | Depression symptoms | Internalizing symptoms | *r* = -.24^**^ | *r* = -.24^**^  [-.38, -.10] |  |  | Morningness preference was associated with lower depression symptoms over time. |
| Itani et al., 2018 | Poor sleep quality  (S) | Mental health status | Internalizing symptoms | Early adolescents:  Poor sleep quality vs good or normal: OR: 3.72  [0.91-15.15];  Late adolescents: Poor sleep quality vs good or normal:  OR 1.83^*^  [1.05 – 3.19] |  |  |  | Poor sleep quality was associated with a higher risk of internalizing symptoms over time in late adolescents. |
| Jiang et al., 2022 * | Symptoms of insomnia  (S) | Resilience | Psychological well-being |  |  | *r* = -.31^***^ | *r* = .31^***^  [.18, .44] | Higher levels of insomnia symptoms were associated with lower levels of psychological well-being over time. |
| Kalak et al., 2014 * | Sleep duration  (S) | Psychological well-being | Psychological well-being | 10–11-year-olds:  *r* = .11^*^;  12–13-year-olds:  *r* = 0.12^**^;  14–15-year-olds:  *r* =.12^**^ | 10–11-year-olds:  *r* = .11^*^  [.04, .18]  12–13-year-olds:  *r* = 0.12^**^  [.5, .19]  14–15-year-olds:  *r* =.12^**^  [.5, .19] | 11–12-year-olds:  *r* =.04;  12–13-year-olds:  *r* =.10^**^;  13–14-year-olds:  *r* =.10^*^ | 11-12-year-olds:  *r* =.04  [-.03, .11];  12-13-year-olds:  *r* =.10^**^  [.03, .17];  13-14-year-olds:  *r* =.10^*^ | Longer sleep duration was bidirectionally associated with higher levels of psychological well-being over time. The association was not bidirectional only in the 10-11 years old group. |
| Kaneita et al., 2009 | Symptoms of insomnia  (S) | Mental health status | Internalizing symptoms | Sleep problems at baseline and follow-up:  OR 4.76^**^  [2.44 – 9.29] |  |  |  | Insomnia symptoms from baseline to follow-up were associated with a higher risk of internalizing symptoms over time. |
| Kechter & Leventhal, 2019 * | Symptoms of insomnia  (S) | Internalizing symptoms | Internalizing symptoms |  |  | *r* = .21^***^ | *r* = -.21^***^  [-.25, -.17] | Higher levels of insomnia symptoms were associated with higher levels of internalizing symptoms over time. |
| Kenny et al., 2016 | Sleep duration  (S) | Positive and negative emotions | Subjective well-being | n/a | n/a | n/a | n/a | Adolescents who did not get sufficient sleep reported higher average daily sadness, anger, and worry and lower daily happiness. |
| Kortesoja et al., 2020 * | Sleep duration; symptoms of insomnia  (S) | Emotional difficulties; conduct problems; attention problems | Internalizing symptoms; externalizing symptoms | Sleep duration T1 – Internalizing symptoms T2:  *r* = -.11^**^  Insomnia symptoms T1- Internalizing symptoms T2:  *r* = .18^**^  Sleep duration T1 – Attention problems T2:  *r* = -.07^**^  Insomnia symptoms T1– Attention problems T2:  *r* = -.14^**^  Sleep duration T1 – Conduct problems T2:  *r* = -.07^**^  Insomnia symptoms T1– Conduct problems T2:  *r* = .06^**^ | Sleep duration T1 – Internalizing symptoms T2:  *r* = -.11^**^  [-.16, -.06]  Insomnia symptoms T1- Internalizing symptoms T2:  *r* = -.18^**^  [-.23, -.13]  Sleep duration T1 – Attention problems T2:  *r* = -.07^**^  [-.10, -.04]  Insomnia symptoms T1– Attention problems T2:  *r* = -.14^**^  [-.19, -.09]  Sleep duration T1 – Conduct problems T2:  *r* = -.07^**^  [-.10, -.04]  Insomnia symptoms T1– Conduct problems T2:  *r* = -.06^**^  [-.11, -.01] | Internalizing symptoms T1 – Sleep duration T2:  *r* = -.08^**^  Internalizing symptoms T1 – Sleep problems T2:  *r* = .19^**^  Attention problems T1 – Sleep duration T2:  *r* = -.07^**^  Attention problems T1 – Sleep problems T2:  *r* = -.21^**^  Conduct problems T1 –Sleep duration T2:  *r* = -.07^**^  Conduct problems T1 –Sleep problems T2:  *r* = .06^**^ | Internalizing symptoms T1 – Sleep duration T2:  *r* = -.08^**^  [-.11, -.05]  Internalizing symptoms T1 – Sleep problems T2:  *r* = -.19^**^  [-.22, -.16]  Attention problems T1 – Sleep duration T2:  *r* = -.07^**^  [-.10, -.04]  Attention problems T1 – Sleep problems T2:  *r* = -.21^**^  [-.26, -.16]  Conduct problems T1 –Sleep duration T2:  *r* = -.07^**^  [-.10, -.04]  Conduct problems T1 –Sleep problems T2:  *r* = -.06^**^  [-.11, -.01] | Shorter sleep duration and higher symptoms of insomnia were bidirectionally associated with internalizing and externalizing symptoms over time. |
| Kuo et al., 2015 * | Sleep duration  (S) | Depression symptoms; risky behavior | Internalizing symptoms; externalizing symptoms | Depression symptoms:  *r* = .06  Risky behaviors:  *r* = -.04 | Depression symptoms:  *r* = .06  [-.07, .19]  Risky behaviors:  *r* = -.04  [-.17, .09] |  |  | Sleep duration was not associated with internalizing and externalizing symptoms over time. |
| Latina et al., 2021 * | Symptoms of insomnia  (S) | Depression symptoms; self-harm | Internalizing symptoms | Depression symptoms:  *r* = .36^***^  Self-harm:  *r* =.16^***^ | Depression symptoms:  *r* = -.36^***^  [-.41, -.31]  Self-harm:  *r* = -.16^***^  [-.21, -.11] | Depression symptoms:  *r* =41^***^  Self-harm:  *r* = .22^***^ | Depression symptoms:  *r* = -.41^***^  [-.45, -.37]  Self-harm:  *r* = -.22^***^  [-.27, -.17] | Higher symptoms of insomnia were bidirectionally related to higher internalizing symptoms over time. |
| Leonard et al., 2021 * | Sleep quality (Sleep onset latency)  (S) | Attention control difficulties | Externalizing symptoms | *r* =.23^***^ | *r* = -.23^***^  [-.33, -.13] |  |  | Lower sleep quality was associated with higher externalizing symptoms over time. |
| Lepore & Kliewer, 2013 * | Symptoms of insomnia  (S) | Depression symptoms | Internalizing symptoms |  |  | *r* = .32^***^ | *r* = -.32^***^  [-.50, -.24] | Higher symptoms of depression were associated with higher insomnia symptoms over time. |
| Li et al., 2021 * | Poor sleep quality; Symptoms of insomnia;  Circadian preference  (S) | Depression and anxiety symptoms | Internalizing symptoms | Poor sleep quality T1 – Depression T2: *r* =.31^***^  Poor sleep quality T1 – Anxiety T2:  *r* =.27^***^  Insomnia symptoms T1 – Depression symptoms T2:  *r* = .41^***^  Insomnia symptoms T1 – Anxiety T2:  *r* = .37^***^ | Poor sleep quality T1 – Depression T2:  *r* = -.31^***^  [-.37, -.25]  Poor sleep quality T1 – Anxiety T2:  *r* = -.27^***^  [-.33, -.21]  Insomnia symptoms T1 – Depression symptoms T2:  *r* = -.41^***^  [-.47, -.35]  Insomnia symptoms T1 – Anxiety T2:  *r* = -.37^***^  [-.43, -.31] | Depression T1 – Poor sleep quality T2:  *r* =.38^***^  Anxiety T1 – Poor sleep quality T2:  *r* =.36^***^  Depression T1 – Insomnia symptoms T2:  *r* =.50^***^  Anxiety T1 – Insomnia symptoms T2:  *r* =.46^***^ | Depression T1 – Poor sleep quality T2:  *r* = -.38^***^  [-.44, -.32]  Anxiety T1 – Poor sleep quality T2:  *r* = -.36^***^  [-.42, -.30]  Depression T1 – Insomnia symptoms T2:  *r* = -.50^***^  [-.55, -.45]  Anxiety T1 – Insomnia symptoms T2:  *r* = -.46^***^  [-.51, -.41] | Poor sleep quality and high levels of insomnia symptoms were bidirectionally associated with higher internalizing symptoms. Moreover, no significant association over time was found between circadian preference and internalizing symptoms over time. |
| Lin et al., 2022 * | Sleep duration, poor sleep quality  (S) | Depression and anxiety symptoms | Internalizing symptoms | Sleep duration T1 - Depression T2:  *r* = -.26^***^  Poor sleep quality T1 - Depression T2: *r* = .50^***^  Sleep duration T2 - Anxiety T2:  *r* = -.24^***^  Poor sleep quality T1 - Anxiety T2:  *r* = .42^***^ | Sleep duration T1 - Depression T2:  *r* = -.26^***^  [-.38, -.14]  Sleep quality T1 - Depression T2:  *r* = -.50^***^  [-.60, -.40]  Sleep duration T2 - Anxiety T2:  *r* = -.24^***^  [-.36, -.12]  Sleep quality T1 - Anxiety T2:  *r* =-.42^***^  [-.42, -.53] | Depression T1- Sleep duration T2:  *r* = -.20^***^  Depression T1 – Poor sleep quality T2:  *r* = .51^***^  Anxiety T1 - Sleep duration T2:  *r* = -.17^***^  Anxiety T1 - Sleep quality T2:  *r* =.45^***^ | Depression T1- Sleep duration T2:  *r* = -.20^***^  [-.32, -.08]  Depression T1 – Poor sleep quality T2:  *r* = -.51^***^  [-.60, -.42]  Anxiety T1 - Sleep duration T2:  *r* = -.17^***^  [-.29, -.05]  Anxiety T1 – Poor sleep quality T2:  *r* = -.45^***^  [-.55, -.35] | Poor sleep quality and short sleep duration were bidirectionally associated with higher internalizing symptoms over time. |
| Liu et al., 2021(a)* | Poor sleep quality (S) | Cyberbullying perpetration; emotional distress | Internalizing and externalizing symptoms | Bullying:  *r* = .27^***^    Emotional distress:  *r* =.26^***^ | Bullying:  *r* = -.27^***^  [-.34, -.20]    Emotional distress:  *r* = -.26^***^  [-.33, -.19] | Bullying:  *r* = .40^***^  Emotional distress: *r* =.41^***^ | Bullying:  *r* = -.40^***^  [-.46, -.34]  Emotional distress:  *r* = -.41^***^  [-.43, -.39] | Poor sleep quality was associated with higher levels of internalizing and externalizing symptoms over time. |
| Liu et al., 2021(b) | Daytime sleepiness  (S) | Self-harm | Internalizing symptoms | Repeated self-harm:  OR: 1.16  [0.45-2.95] |  |  |  | No association was found between daytime sleepiness and self-harm over time. |
| Liu et al., 2023 | Symptoms of insomnia  (S) | Substance use | Externalizing symptoms | OR: 2.00^*^  [1.26–3.19] |  |  |  | Higher symptoms of insomnia were associated with higher risk of substance use over time. |
| Lovato et al., 2017 | Sleep duration Sleep quality  (S) | Depressed mood | Internalizing symptoms | n/a | n/a | n/a | n/a | No associations were found between sleep quality, sleep duration and depression symptoms over time. |
| Lundh et al., 2013 * | Symptoms of insomnia  (S) | Self-harm | Internalizing symptoms | Females: *r* = .13^*^  Males: *r* =.40^**^ | Females: *r* = -.13^*^  [-.22, -.04]  Males: *r* = -.40^**^  [-.48, -.32] | Females: *r* = .10  Males: *r* =.32^**^ | Females:  *r* = -.10  [-.17, -.03]  Males:  *r* = -.32^**^  [-.40, -.27] | Higher symptoms of insomnia were bidirectionally associated to higher internalizing symptoms in males over time. In females an association between higher insomnia symptoms and internalizing symptoms over time was found but not vice versa. |
| Luo et al., 2013 | Symptoms of insomnia  (S) | Depression and anxiety symptoms | Internalizing symptoms |  |  | Depression:  OR: 1.47^*^  [1.07,2.03]  Anxiety:  1.48^*^  [1.08-2.03] |  | Depression and anxiety symptoms were associated with higher risk of insomnia symptoms over time. |
| Marino et al., 2022 * | Symptoms of insomnia  (S) | Depression symptoms | Internalizing symptoms | *r* = .07^*^ | *r* = -.07^*^  [-.13, -.01] | *r* = .10^**^ | *r* = -.10^**^  [-.16, -.04] | Higher symptoms of insomnia were bidirectionally associated with higher depression symptoms over time. |
| Matsumoto et al., 2021 | Poor sleep quality (S) | Poor mental health | Internalizing symptoms |  |  | OR: 3.10^*^  [1.76 -5.47] |  | Poor mental health status was associated with higher risk for poor sleep quality over time. |
| Maume, 2017 | Sleep duration  (S) | Depression  symptoms | Internalizing symptoms | n/a | n/a | n/a | n/a | Sleep duration was associated with reporting depressive symptoms. |
| Nowakowski et al., 2014 * | Sleep duration  (S) | Depression  symptoms | Internalizing symptoms | *r* = -.23^***^ | *r* = -.23^***^  [-.29, -.17] |  |  | Longer sleep duration was associated with lower internalizing symptoms over time. |
| Roberts & Duong, 2013 | Symptoms of insomnia  (S) | Depression  symptoms | Internalizing symptoms | OR: 2.57^*^  [1.33 – 4.98] |  | OR: 1.94^*^  [1.06 – 3.54] |  | Higher levels of insomnia symptoms were bidirectionally associated with a high risk of internalizing symptoms over time. |
| Roberts & Duong, 2014 | Sleep duration  (S) | Depression  symptoms | Internalizing symptoms | OR: 5.21^*^  [2.48 – 10.93] |  | OR: 5.36 ^*^  [2.91 – 9.89] |  | Short sleep duration was bidirectionally associated with a high risk of internalizing symptoms over time. |
| Roberts et al., 2002 | Symptoms of insomnia  (S) | Self-esteem | Psychological well-being | OR: 3.78^***^  [2.89 – 4.94] |  |  |  | Higher levels of insomnia symptoms were associated with a high presence of self-esteem over time. |
| Rosen et al., 2021 * | Sleep duration  (S) | General internalizing and externalizing symptoms | Internalizing and externalizing symptoms | Internalizing  *r* = -.02  Externalizing  *r* = -.13 | Internalizing  *r* = -.02  [-.17, .12]  Externalizing  *r* = -.13  [-.31, .05] |  |  | No association was found between sleep duration and internalizing and externalizing symptoms over time. |
| Scherrer & Preckel, 2021 | Chronotype (Morningness)  (S) | Self-efficacy | Psychological well-being | *r* = .15^**^ | *r* = .15^**^  [.01, .29] |  |  | The morning circadian preference was associated with higher self-efficacy over time. |
| Sladek et al., 2019 * | Sleep duration  (S) | Depression symptoms | Internalizing symptoms | *r* = -.04 | *r* = -.04  [-.29, .21] |  |  | No association was found between sleep duration and depression symptoms over time. |
| Soffer-Dudek & Sadeh, 2013* | Sleep quality (Sleep efficiency)  (S) | General internalizing and externalizing symptoms | Internalizing and externalizing symptoms | Internalizing:  *r* = .02  Externalizing  *r* = -.07 | Internalizing:  *r* = .02  [-.22, .26]  Externalizing  *r* = -.07  [-.31, .17] | Internalizing:  *r* = .07  Externalizing  *r* = -.07 | Internalizing:  *r* = .07  [-.17, .31]  Externalizing  *r* = -.07  [-.31, .17] | There was not a bidirectional association between sleep quality and internalizing and externalizing symptoms over time. |
| Thorburn-Winsor et al., 2022 * | Sleep duration; Bedtime  (O) | Depression symptoms | Internalizing symptoms | Sleep duration  *r* = -.04  Bedtime  *r* = .04 | Sleep duration  *r* = -.04  [-.12, .04]  Bedtime  *r* = -.04  [-.12, .04] |  |  | No association was found between sleep duration and bedtime at one time and internalizing symptoms at a later time. |
| Troxel et al., 2019 * | Sleep duration during weekday;  Symptoms of insomnia  (S) | Mental health status;  Risky behaviors | Internalizing and externalizing symptoms | Sleep duration T1 – Internalizing T2:  *r* = .06  Insomnia symptoms T1 – Internalizing T2:  *r* = .25^***^  Sleep duration T1 – Externalizing T2:  *r* = -.04  Insomnia symptoms T1 – Externalizing T2:  *r* = .07^**^ | Sleep duration T1 – Internalizing T2:  *r* = .06  [.02, .11]  Sleep problems T1 – Internalizing T2:  *r* = -.25^***^  [-.29, -.20]  Sleep duration T1 – Externalizing T2:  *r* = -.04  [-.09, .00]  Insomnia symptoms T1 – Externalizing T2:  *r* = -.07^**^  [-.13, -.03] | Internalizing T1 – Sleep duration T2:  *r* = -.10^***^  Internalizing T1 – Insomnia symptoms T2:  *r* = .23^***^ | Internalizing T1 – Sleep duration T2:  *r* = -.10^***^  [-.15, -.06]  Internalizing T1 – Insomnia symptoms T2:  *r* = -.23^***^  [-.27, -.19] | Higher insomnia symptoms and short sleep duration were bidirectionally related to higher internalizing symptoms over time. Moreover, higher insomnia symptoms, but not shorter sleep duration were bidirectionally associated with higher externalizing symptoms over time. |
| Tu & Cai, 2020 * | Symptoms of insomnia  (S) | Depression symptoms  (S) | Internalizing symptoms |  |  | *r* = .26* | *r* = .26*  [-.46, -.06] | Higher depression symptoms were associated with higher insomnia symptoms over time. |
| Tu et al., 2019 * | Sleep quality (S) | Depression and anxiety symptoms | Internalizing symptoms |  |  | Anxiety:  *r* = -.30^**^  Depression:  *r* = .31^**^ | Anxiety:  *r* = -.30^**^  [-.48, -.12]  Depression:  *r* = .31^**^  [.13, .49] | Higher anxiety symptoms were associated with lower sleep quality over time. Moreover, higher depression symptoms were associated to higher sleep quality over time. |
| Vaszonyi et al., 2021 * | Sleep duration, sleep quality  (S) | Externalizing symptoms; depression; anxiety; subjective well-being; self esteem | Internalizing and externalizing symptoms; psychological well-being; subjective well-being | Sleep quality T1 – Depression T2:  *r* = -.21^**^  Sleep quality T1 – Anxiety T2:  *r* = -.26^**^  Sleep quality T1 – Low self-esteem T2:  *r* = -.22^**^  Sleep quality T1 – Low well-being T2:  *r* = -.14^*^  Sleep quality T1 – Externalizing symptoms T2:  *r* = -.21^**^  Sleep duration T1 – DepressionT2:  *r* =.09  Sleep duration T1 – Anxiety T2:  *r* = -.04  Sleep duration T1 – Low self-esteem T2:  *r* = .02  Sleep duration T1 – Low well-being T2:  *r* = .06  Sleep duration T1 – Externalizing symptoms T2:  *r* = -.01 | Sleep quality T1 – Depression T2:  *r* = -.21^**^  [-.30, -.12]  Sleep quality T1 – Anxiety T2:  *r* = -.26^**^  [-.35, -.17]  Sleep quality T1 – Low self-esteem T2:  *r* = -.22^**^  [.13, .31]  Sleep quality T1 – Low well-being T2:  *r* = .14^*^  [.05, .23]  Sleep quality T1 – Externalizing symptoms T2:  *r* = -.21^**^  [-.30, -.12]  Sleep duration T1 – DepressionT2:  *r* =.09  [-.00, .18]  Sleep duration T1 – Anxiety T2:  *r* = -.04  [-.13, .05]  Sleep duration T1 – Low self-esteem T2:  *r* = .02  [-.07, .11]  Sleep duration T1 – Low well-being T2:  *r* = .06  [-.03, .15]  Sleep duration T1 – Externalizing symptoms T2:  *r* = -.01  [-.10, -.08] |  |  | Higher sleep quality was associated with depression, anxiety, low self-esteem, lower well-being and lower externalizing symptoms over time. The same association was not found for sleep duration. |
| Vaszonyi et al., 2022 * | Sleep quality  (S) | Depression and anxiety symptoms | Internalizing symptoms | Females:  Depression:  *r* = -.37^**^  Anxiety  *r* = -.34^**^  Males:  Depression:  *r* = -.37^**^  Anxiety:  *r* = -.18^**^ | Females:  Depression:  *r* = -.37^**^  [-.46, -.28]  Anxiety  *r* = -.34^**^  [-.44, -.24]  Males:  Depression:  *r* = -.18^**^  [-.30, -.06]  Anxiety:  *r* = -.21^**^  [-.33, -.09] | Females:  Depression:  *r* = -.35^**^  Anxiety  *r* = -.39^**^  Males:  Depression:  *r* = -.37^**^  Anxiety:  *r* = -.32^**^ | Females:  Depression:  *r* = -.35^**^  [-.44, -.26]  Anxiety  *r* = -.39^**^  [-.48, -.30]  Males:  Depression:  *r* = -.37^**^  [-.48, -.26]  Anxiety:  *r* = -.32^**^  [-.44, -.20] | Higher sleep quality was associated bidirectionally with lower depression and anxiety symptoms over time. |
| Vernon et al., 2017* | Symptoms of insomnia  (S) | Depression symptoms;  Externalizing symptoms | Internalizing and externalizing symptoms | Externalizing symptoms:  *r* = .11^**^  Depression symptoms  *r* = .36^**^ | Externalizing symptoms:  *r* = -.11^**^  [-.18, -.04]  Depression symptoms  *r* = -.36^**^  [-.42, -.30] | Externalizing symptoms:  *r* = -.15^**^  Depression symptoms:  *r* = .32^**^ | Externalizing symptoms:  *r* = -.15^**^  [-.21, -.09]  Depression symptoms:  *r* = -.32^**^  [-.38, -.26] | Higher symptoms of insomnia were bidirectionally associated to higher internalizing and externalizing symptoms over time. |
| Vernon et al., 2018 * | Sleep quality  (S) | Self-esteem | Psychological well-being | Sleep quality:  *r* = .23^*^ | Sleep quality:  *r* = .23^*^  [.17, .29] | Sleep quality:  *r* = .32^*^ | Sleep quality:  *r* = .32^*^  [.26, .38] | Higher sleep quality was bidirectionally associated with higher psychological well-being over time. |
| Wang et al., 2020 * | Poor sleep quality  (S) | Resilience | Psychological well-being | *r* = -.21^*^ | *r* = .21^*^  [.15, .27] | *r* = -.23^*^ | *r* = .23^*^  [.17, .29] | Poor sleep quality was bidirectionally associated with lower psychological well-being over time. |
| Wang & Yip, 2020 * | Sleep duration  (S) | Life satisfaction, positive and negative emotions | Subjective well-being | Life satisfaction:  *r* = .04  Positive emotions:  *r* = .04  Negative emotions  *r* = -.11^***^ | Life satisfaction:  *r* = .04  [-.08, .16]  Positive emotions:  *r* = .04  [-.08, .16]  Negative emotions  *r* = -.11^***^  [-.01, .23] |  |  | Longer sleep duration was associated with negative emotions over time, but not with life satisfaction and positive emotions. |
| Wong & Brower, 2012 | Symptoms of insomnia  (S) | Suicidal intention | Internalizing symptoms | OR: 1.95^*^  [1.41 – 2.69] |  |  |  | Higher symptoms of insomnia were associated with a higher risk of suicidal intention over time. |
| Wong et al., 2015 | Symptoms of insomnia  (S) | Substance use | Externalizing symptoms | OR: 1.07  [0.99 – 1.61] |  |  |  | No association was found between symptoms of insomnia and externalizing symptoms over time. |
| Yip et al., 2022* | Symptoms of insomnia  (S) | Anxiety symptoms; positive and negative affect | Internalizing symptoms; Subjective well-being | Anxiety:  *r* =.01  Negative affect:  *r* =.01  Positive affect:  *r* = .01 | Anxiety:  *r* = -.01  [-.20, .18]  Negative affect:  *r* = -.01  [-.20, .18]  Positive affect:  r= -.01  [-.20, .18] | Anxiety:  *r* = .08  Negative affect:  *r* =.44^***^  Positive affect:  *r* = .15 | Anxiety:  *r* = .08  [-.26, .42]  Negative:  *r* =.44^***^  [-.55, -.33]  Positive:  *r* = .15  [-.48, .18] | An association between negative affect and insomnia symptoms was found over time, but not between insomnia symptoms, anxiety and positive affect. |
| Zeiders, 2017 * | Sleep duration  (S) | Internalizing symptoms | Internalizing symptoms | *r* = .06 | *r* = .06  [-.16, .28] | *r* = -.22^*^ | *r* = -.22^*^  [-.43, -.01] | Higher internalizing symptoms were associated with shorter sleep duration over time, but not vice versa. |
| Zhang et al., 2022 | Sleep duration  (S) | Depression and anxiety symptoms | Internalizing symptoms | Depression:  OR: 2.81  [1.66 - 4.75]  Anxiety:  OR: 3.07  [1.87 - 5.07] |  |  |  | Shorter sleep duration (less than 8 hours) was associated with a higher risk to report depression and anxiety symptoms. |
|  |  |  |  |  |  |  |  |  |

*Notes*. ^1^Cross-lagged effects between sleep at one time point and mental health and positive well-being variables measured at the last time point considered in the study. ^2^Cross-lagged effects between mental health and positive well-being variables measured at one time point and sleep measured the last time point considered in the study. (S) = subjective assessment of sleep health parameters; (O) = objective assessment of sleep health parameters. *r* = Pearson’s correlation (confidence intervals are reported between square brackets); *ρ* = Spearman’ rho; OR= Odds ratio and confidence interval in parenthesis. ^***^*p* <.001, ^**^*p* < .01, ^*^*p* < .05. Studies marked with an asterisk (*) are those included in the meta-analyses reported in Table 2 (when the effect sizes could not be converted into Pearson’s correlations, it was not possible to include them in the meta-analytic computations). To compute the overall meta-analytic summary, the effect sizes of studies were recoded so that longer sleep duration, higher sleep quality, and lower levels of insomnia symptoms at T1 were related to lower internalizing and externalizing symptoms and higher subjective and psychological well-being at T2.

**Document S7: Forest plot for the meta-analysis on the longitudinal association between Sleep at T1 and Internalizing symptoms T2**


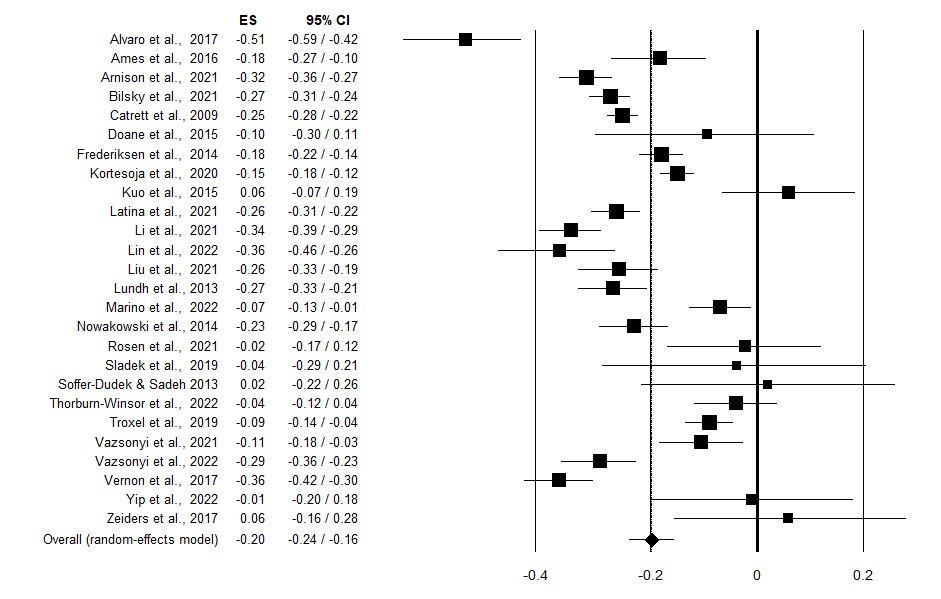


**Document S8: Forest plot for the meta-analysis on the longitudinal association between Internalizing symptoms T1 and Sleep T2**


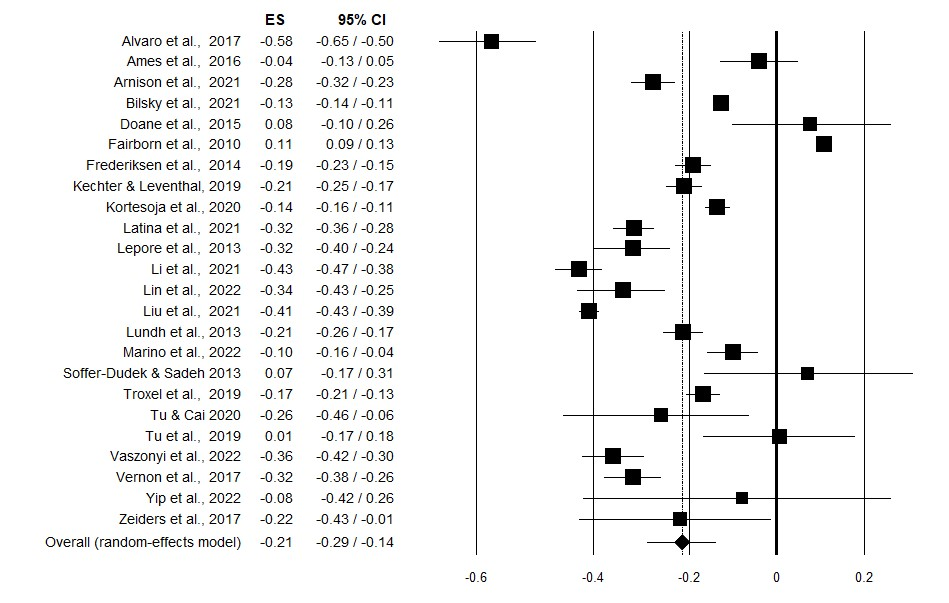


**Document S9: Forest plot for the meta-analysis on the longitudinal association between Sleep at T1 and Externalizing symptoms T2**


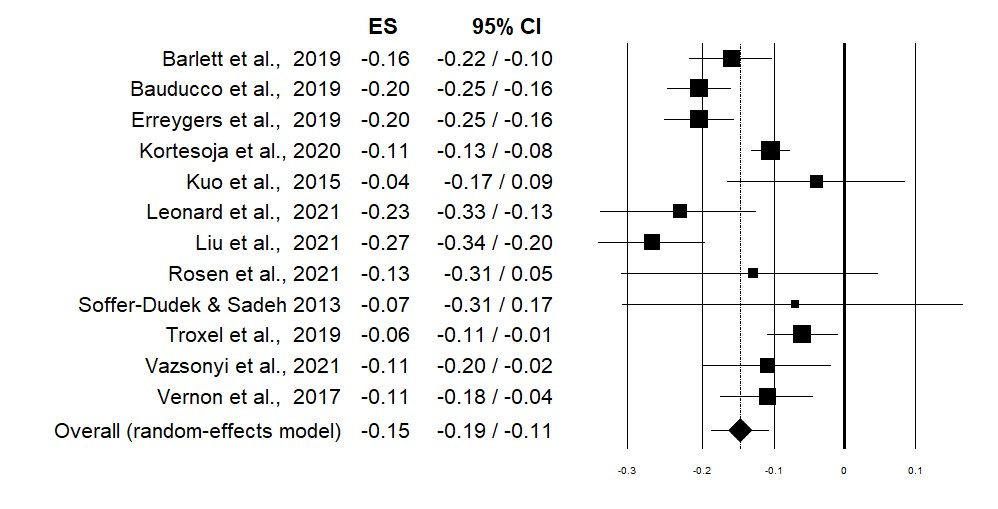


**Document S10: Forest plot for the meta-analysis on the longitudinal association between Externalizing symptoms T1 and Sleep T2**


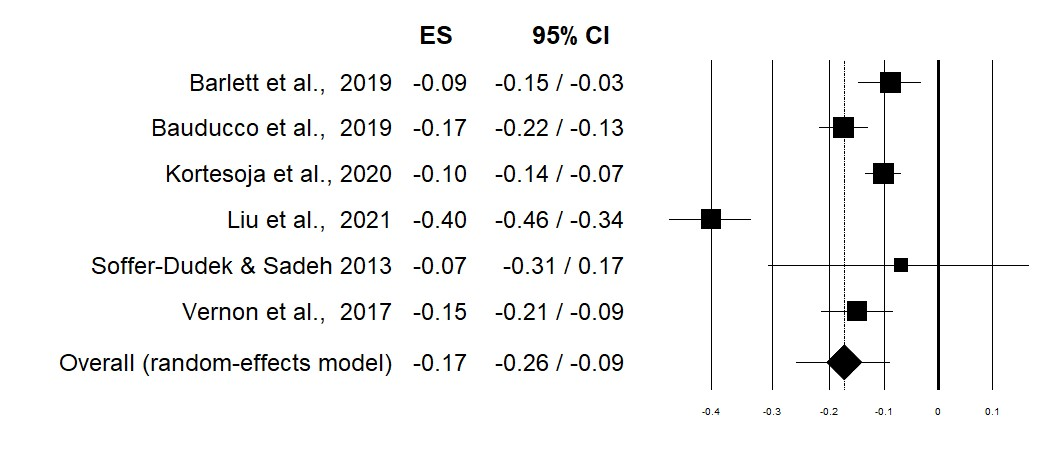


**Document S11: Forest plot for the meta-analysis on the longitudinal association between Sleep at T1 and Subjective well-being T2**


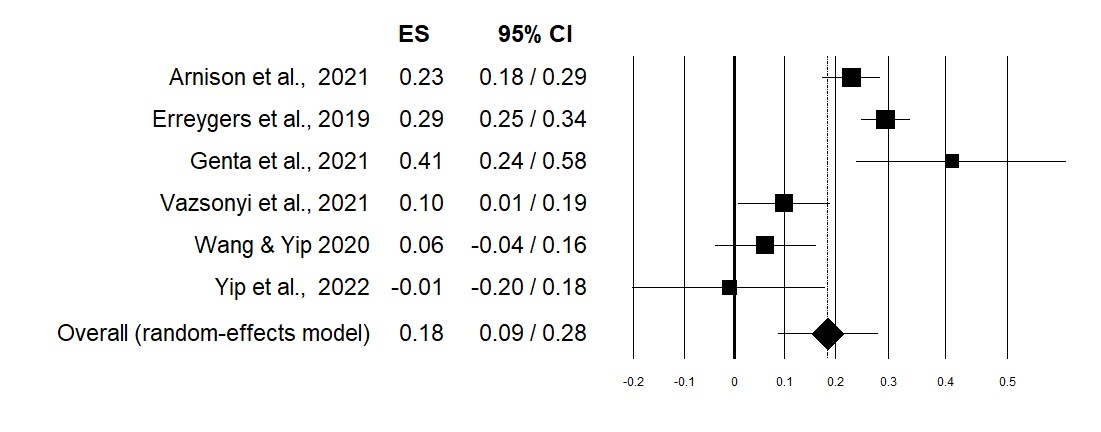


**Document S12: Forest plot for the meta-analysis on the longitudinal association between Sleep at T1 and Psychological well-being T2**


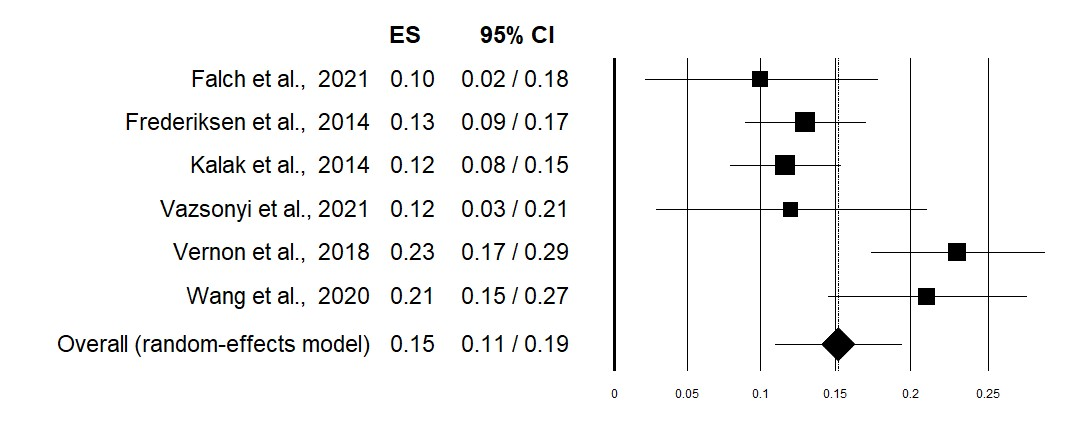


**Document S13: Forest plot for the meta-analysis on the longitudinal association between Psychological well-being at T1 and Sleep T2**


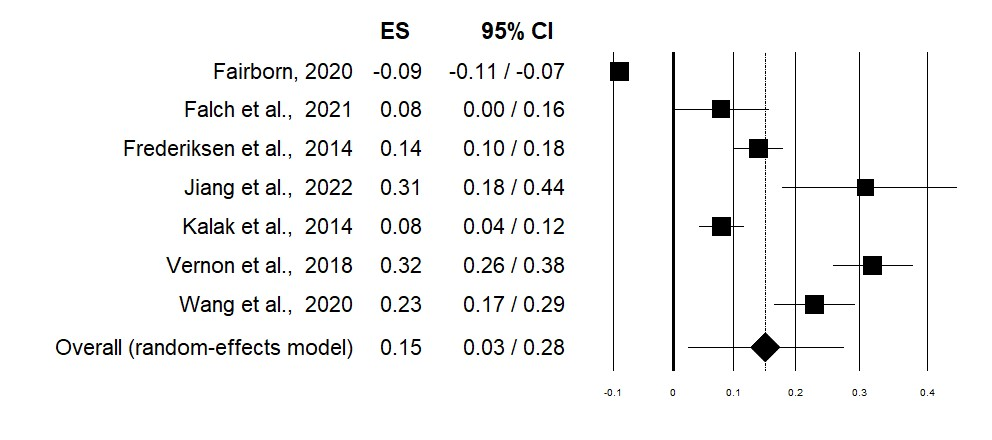

Supplement: Supplementary file 1 [file mmc1.docx]
